# Two Short Repeats in the 5′ Untranslated Region of Insulin-like Androgenic Gland Factor in *Procambarus clarkii* (*PcIAG*) That Regulate *PcIAG* Expression

**DOI:** 10.3390/ijms231810348

**Published:** 2022-09-07

**Authors:** Siqi Yang, Rong Sun, Xiuli Chen, Qishuai Wang, Pengfei Feng, Yongzhen Zhao, Yanhe Li

**Affiliations:** 1 College of Fisheries, Key Laboratory of Freshwater Animal Breeding, Ministry of Agriculture and Rural Affair, Huazhong Agricultural University, Wuhan 430070, China; 2 Engineering Research Center of Green Development for Conventional Aquatic Biological Industry in the Yangtze River Economic Belt, Ministry of Education, Huazhong Agricultural University, Wuhan 430070, China; 3 Guangxi Shrimp Breeding Engineering Technology Research Center, Guangxi Key Laboratory of Aquatic Genetic Breeding and Healthy Aquaculture, Guangxi Academy of Fishery Sciences, Nanning 530021, China

**Keywords:** *Procambarus clarkii*, *IAG*, repeated sequences, RNAi, exosomes, mRNA, miRNA

## Abstract

Insulin-like androgenic gland factor (*IAG*) plays an important role in sex manipulation in decapods. Understanding the molecular regulation mechanism of *IAG* in *Procambarus clarkii* (*PcIAG*) is important for realizing its sex control. In this study, the promoter and gene structure of *PcIAG*, mRNA, and miRNA expression profiles after interfering with two siRNAs synthesized according to the two short repeats in the 5′ untranslated regions (5′UTR) of *PcIAG* were analyzed, and miRNAs of exosomes were investigated to explore the role of repeated sequences with tandem two short repeats located in the 5′UTR of *PcIAG* isolated from the androgenic gland (AG) in the regulation of *IAG* expression. The results showed that the repeated sequences of 5′UTR only occurred completely in the cDNA from AG, and the function of the two repeats was different in regulating the expression of *PcIAG*, in which the Wnt signaling pathway may be involved. Furthermore, we found that six miRNAs including miR-133, miR-193, miR-34, miR-1, miR-100, and let-7 might be involved in the regulation of the expression of *PcIAG*, wherein miR-133 might directly be related with the repeated sequences of 5′UTR.

## 1. Introduction

*Procambarus clarkii* is an important aquatic commercial crayfish in China [1]. The meat content of female *P. clarkii* is higher than that of the male in the same weight condition, which means the morphology of *P. clarkii* has sexual dimorphism [2]. So, understanding its sex manipulation mechanism will help promote its industrial development [3]. The androgenic gland (AG) can regulate male differentiation and primary and secondary characteristics, which plays a key role in sex manipulation in crustaceans [4,5,6]. And the androgenic gland hormone as a key active substance in the function of AG might be encoded by the insulin-like androgenic gland hormone gene (*IAG*) in Decapoda [7]. Ventura et al. [5] obtained fully functionally reversed neofemales of *Macrobrachium rosenbergii* successfully through silencing *IAG*. Rosen et al. [8] provided evidence that silencing the *IAG* gene in intersex *Cherax quadricarinatus* feminizes male-related phenotypes. A long-term silencing of a putative *IAG* receptor gene (*FcIAGR*) in *Fenneropenaeus chinensis* led to most of the germ cells in the testis being arrested at the secondary spermatocytes [9]. Male Chinese mitten crab (*Eriocheir sinensis*) being transformed into neo-females after inhibiting *Es-IAG* production [10]. Liu et al. [11] found that *IAG* gene knockdown in peppermint shrimp *Lysmata vittata* induced a retarded development of male gonopores.

With the development of sequencing technology, mRNA and miRNA sequencing have become important means to identify sex-related genes and understand the molecular regulatory mechanisms of gender-related genes [3,12,13]. Jin et al. [14] analyzed the expression profiles of mRNAs and miRNAs during the sex-differentiation sensitive period in *Macrobrachium nipponense*, a total of three sex-related miRNAs and five important sex-related metabolic pathways were also identified. Jiang et al. [3] analyzed the transcriptome of the ovary and testis of *P. clarkii* and identified sex-related genes such as *Vitellogenin*, *cyclin B*, *Cyclin-dependent kinases 2*, *Dmc1,* and *ubiquitin*. And some studies have shown that sRNA can participate in the regulation of gene expression [15]. Orom et al. [16] found that miR-10a interacts with the 5′UTR of mRNA encoding ribosomal proteins to enhance its translation. The 5′untranslated region (5′UTR) plays an important role in the regulation of gene expression [17,18]. However, there are few reports on the regulation of the *IAG* molecular mechanism in *P. clarkii*.

Exosomes are small cell-derived vesicles, which are extensively distributed in various biological fluids [19,20]. Exosomes have multiple activities such as transmitting signals and molecules to other cells [21]. Presently, the research on miRNA in exosomes has been increasing. Sun et al. [22] used next-generation sequencing technology to identify miRNAs in milk exosomes of *Staphylococcus aureus* infected cows and healthy cows and found that 14 known bovine microRNAs were differentially expressed, and bta-miR-142-5p and bta-miR-223 may be potential biomarkers for early detection of bacterial infection in the mammary gland. *IAG* was mainly expressed in AG and regulated by regulatory factors from eye stalk and sex-determining factors, such as sex-lethal (*Sxl*), Doublesex (*Dsx*) and gonad-inhibiting hormone (*GIH*), etc. [7,23]. Exosome miRNA sequencing was performed before and after androgenic gland ablation could help understand the relevant regulatory factors that may be involved in the expression of *IAG*.

In this study, we found that there were two repeats in the 5′UTR of *PcIAG* that might be involved in the regulation of *PcIAG* expression. To explore the role of the two repeats in the regulation of *PcIAG* expression, the promoter and gene structure of *PcIAG*, mRNA, and miRNA expression profiles after interfering with two siRNAs that synthesized according to the two short repeats in 5′UTR of *PcIAG* were analyzed, and miRNA sequencing was performed on exosomes in blood before and after androgenic gland ablation of *P. clarkii*. The results could be expected to provide reference materials at the transcriptional level for the study of the molecular regulation mechanism of *IAG* in crayfish.

## 2. Results

### 2.1. Isolation of the Insulin-like Androgenic Gland Hormone Gene (PcIAG) from Procambarus Clarkii

**Figure 1:**
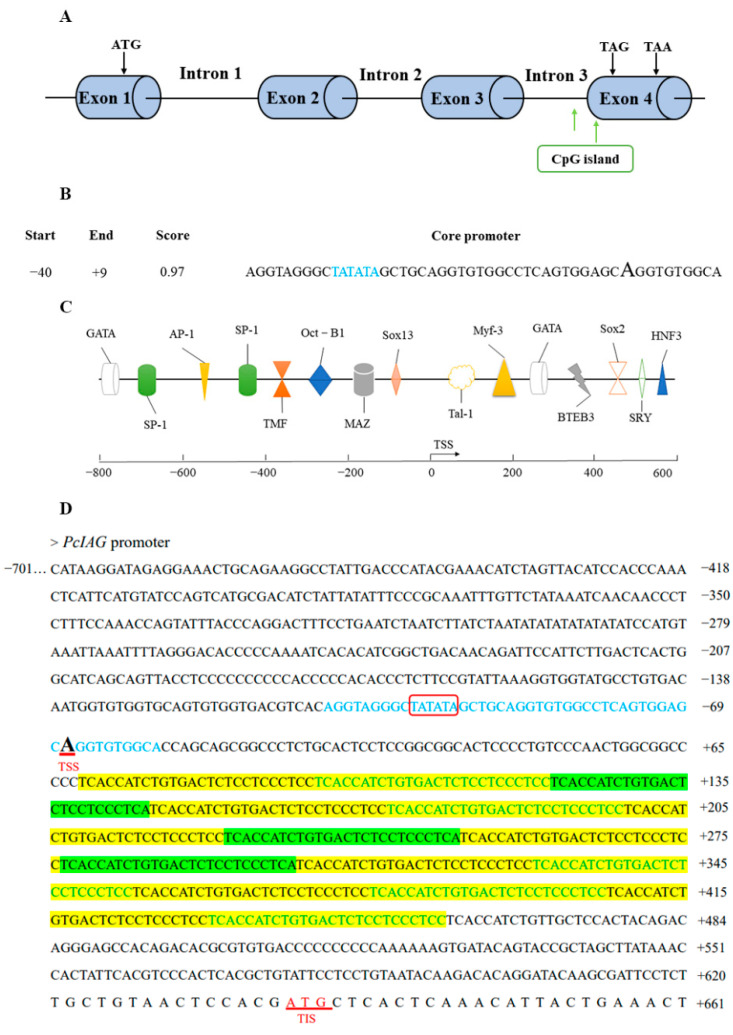
Structural information of *PcIAG* DNA sequence and promoter sequence. (**A**) Structure of the *IAG* gene in *Procambarus clarkii*. Blue box denotes exon (exon1 = 110 bp; exon2 = 116 bp; exon3 = 212 bp; exon4 = 345 bp); the lines middle denotes intron (intron1 = 18992 bp; intron 2 = 103 bp; intron 3 = 1034 bp); green box denotes CpG island (Criteria used: Island size > 100, GC Percent > 50.0, Obs/Exp > 0.6); ATG showed the translation start codon, TAA and TAG showed the stop codons of *PcIAG*. (**B**) The position and score of the core promoter region predicted by the software. TATA box was marked in blue and the transcription start site was shown using an enlarged and bolded character A. (**C**) Predicted a part of transcription factor binding sites on the promoter sequence of *PcIAG*. (**D**) Promoter sequences of *PcIAG*. The two repeats in the repeating region were marked with green and yellow colors, respectively. The core promoter was shown in blue font and the TATA box was circled in red. TSS showed the transcriptional start site and TIS showed the translation start site of *PcIAG*.

The full-length DNA sequences of *PcIAG* consisted of four exons and three introns, with a total length of 20,964 bp (Figure 1A; Figure S1; GenBank: PRJNA727411). As shown in Figure 1A, two CpG islands were predicted by CpGPlot and CpG islands, with a length of 354 bp (located in 20,124–20,477 bp) and 196 bp (located in 20,613–20,809 bp).

The phylogenetic tree of *IAG* DNA sequences of five species showed that *P. clarkii* was the closest similarity to *Procambarus fallax* and *Procambarus virginalis* (Figure S2).

### 2.2. Bioinformatics Analysis of PcIAG Promoter Sequence

The length of the promoter was 1363 bp (Figure S3). More than 400 transcription factor binding sites were predicted, and the transcription factor binding sites main included AP-1, SP-1, OCT-B1, HNF3, GATA, TMF, and sex-related binding sites such as SRY, Sox2, and Sox13 (Figure 1B,C).

As shown in Figure 1D, the repeat sequence region containing two short repeats (TCACCATCTGTGACTCTCCTCCCTCC and TCACCATCTGTGACTCTCCTCCCTCA) was located between the transcription start site and the initiation codon, which indicated that the repeated sequences were located in the 5′UTR.

### 2.3. The Existence Pattern of Repeats in Different Tissues and Developmental Stages of Female and Male Procambarus Clarkii

**Figure 2:**
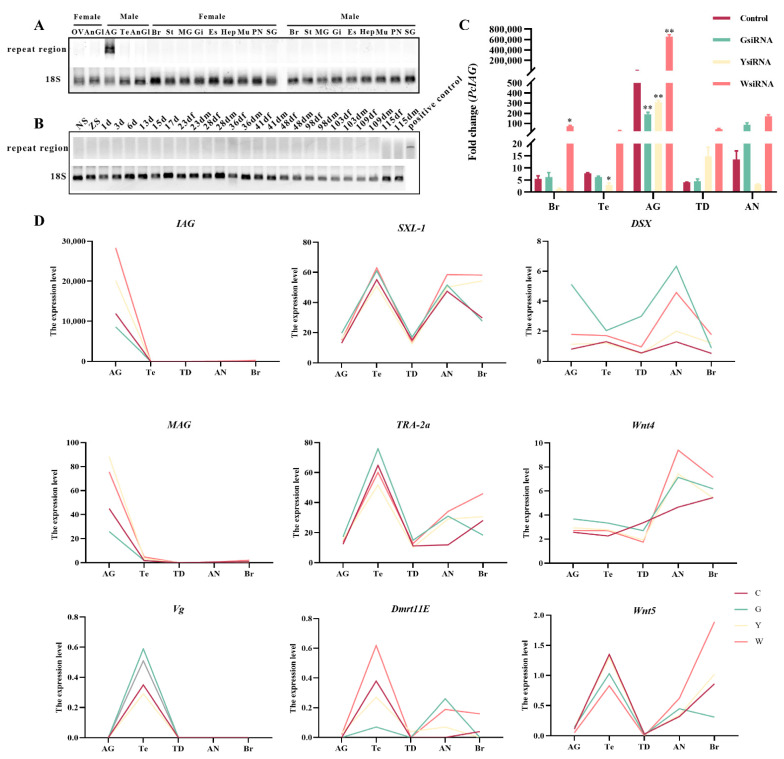
The results of amplification of *PcIAG* repeat regions and the expression of some genes after RNA interference. (**A**) The results of amplification of *PcIAG* repeat regions in male and female *Procambarus clarkii* DNA. AnGl: The antennary glands; Mu: Muscle; Gi: Gill; Es: Eyestalk; Br: Brain; St: Stomach; MG: Midgut; Hep: Hepatopancreas; PN: Periesophageal nerve; SG: Subesophageal ganglia; OV: Ovary; Te: testis; AG: Androgenic gland. (**B**) The results of amplification of *PcIAG* repeat regions in different tissues and different developmental stages of *Procambarus clarkii*. NS: The nauplius stage; ZS: The zoea stage; f: female crayfish; m: male crayfish, 1d–115d: the first to the 115th day after hatching. (**C**) Expression pattern of *IAG* in gonadal and neural tissues after siRNA interference. Br: Brain; Te: Testis; AG: Androgenic gland; TD: Testicular ducts; AN: Abdominal nerve cord. An asterisk represents the significant difference (*p* < 0.05), and two asterisks represent the extremely significant difference (*p* < 0.01). Control denotes the *Procambarus clarkii* injected with saline (control group); GsiRNA denotes the *Procambarus clarkii* injected with 0.02 µg/g body weight GsiRNA (treated group); YsiRNA denotes the *Procambarus clarkii* injected with 0.02 µg/g body weight YsiRNA (treated group); WsiRNA denotes the *Procambarus clarkii* injected with 0.02 µg/g body weight WsiRNA (negative control). (**D**) Effects of siRNA interference on sex-related genes. C denotes the *Procambarus clarkii* injected with saline (control group); G denotes the *Procambarus clarkii* injected with 0.02 µg/g body weight GsiRNA (treated group); Y denotes the *Procambarus clarkii* injected with 0.02 µg/g body weight YsiRNA (treated group); W denotes the *Procambarus clarkii* injected with 0.02 µg/g body weight WsiRNA (negative control).

There were no differences in the repeat sequences between males and females at the DNA level (Figure S4). The *IAG* cDNA sequence from AG tissue contained the region of the repeated sequence, whereas other tissues did not (Figure 2A). There were no products amplified in the cDNA sample of different developmental stages of *P. clarkii* within four months before becoming adult male *P. clarkii* (Figure 2B).

The siRNAs synthesized based on the repeats (Figure 1D) were used to interfere with *PcIAG*. The results showed that the expression level of *PcIAG* was significantly downregulated in the GsiRNA and YsiRNA groups compared with the control group in AG, indicating that GsiRNA and YsiRNA could effectively inhibit the expression of *PcIAG*. In other tissues, GsiRNA and YsiRNA have a poor inhibitory effect or even fail to inhibit the expression of *PcIAG*. WsiRNA could not inhibit the expression of *PcIAG* in the five tissues (Figure 2C). The difference in inhibitory effect might be related to the presence of repeated fragments only in AG tissue (Figure 2).

### 2.4. Analysis of mRNA Expression Profiles in Gonad and Nerve after siRNA Interference

#### 2.4.1. De Novo Assembly and Annotation

A total of 141.98 GB of clean reads was obtained from 20 samples of mRNA libraries sequencing raw data (Table S1). The de novo assembly of the transcripts was performed. After the de novo assembly and removing redundancy, a total of 184,904 unigenes were obtained. The length distribution of the unigenes was between 201 and 21,103 base pairs (bp) with an average length of 719.05 bp (Table S2).

After assembly, the assembled unigenes were annotated functionally based on six databases, including NCBI non-redundant protein sequences (NR), Swiss-prot, Pfam, Clusters of Orthologous Groups of proteins (COG), Gene Ontology (GO), and Kyoto Encyclopedia of Genes and Genomes (KEGG). As a result, 31,400 (16.98%), 20,412 (11.04%), 21,595 (11.68%), 3784 (2.03%), 17,758 (9.60%) and 16,100 (8.71%) unigenes were mapped to NR, Swiss-Prot, Pfam, COG, GO, and KEGG, respectively. A total of 35,083 (18.97%) unigenes were annotated based on these databases (Figure S5).

#### 2.4.2. GO and KEGG Enrichment Analyses of Differentially Expressed Genes

The number of differentially expressed genes (Table S3) in AG tissue of the treatment groups and control group was 2186, 1886 (Figure S6). To better understand the function and classification of differentially expressed genes, GO enrichment and KEGG pathway analyses were performed. The differentially expressed genes between the siRNA interference group and the control group were enriched by many GO terms and KEGG pathways (Table S4; Figure S7).

The KEGG pathway analysis showed that the differentially expressed genes were enriched in seven sex-related pathways, including Ovarian steroidogenesis, Wnt signaling pathway, Progesterone-mediated oocyte maturation, Oocyte meiosis, Estrogen signaling pathway, MAPK signaling pathway, and GnRH signaling pathway (Table S5). These sex-related pathways indicated the *PcIAG* played important role in sex regulation.

In gonadal tissues, differentially expressed genes of the GsiRNA group vs. control group and YsiRNA group vs. control group were enriched in many sex-related GO terms. However, the sex-related GO terms were not enriched in the differentially expressed genes in the WsiRNA group vs. the control group (Table S6). The GO enrichment analysis results indicated that the region of the repeated sequence in the 5′UTR region of *PcIAG* maybe has an effect on sex regulation.

In addition, there were many differences in the GO terms enriched by differentially expressed genes in the GsiRNA group vs. control group and YsiRNA group vs. control group in gonadal tissues. For example, there were GO terms related to the sex-related pathway, Wnt signaling pathway, in differentially expressed genes in the YsiRNA group vs. control group, while the GsiRNA group vs. control group did not (Table S6). There was also a difference in the enriched sex pathways in the GsiRNA group vs. control group and YsiRNA group vs. control group (Table S5). Such could indicate that the regulation of expression of *PcIAG* with GsiRNA and YsiRNA was different although there is only one base difference between the two repeats.

#### 2.4.3. Sex-Related Differentially Expressed Genes

Based on sequence annotation and literature report [3,24,25,26], sex-related genes were identified in mRNA expression profiles (Table S7). The expression levels of *IAG* and membrane-anchored androgenic gland-specific factors (*MAG*) showed similar trends. And the expression of sex-lethal (*Sxl*), Doublesex (*Dsx*), transformer-2a (*TRA-2a*), Wnt family member 4 (*Wnt4*), and Wnt family member 5A (*Wnt5*) increased after *IAG* was inhibited by GsiRNA and YsiRNA in AG tissue (Figure 2D).

### 2.5. Analysis of miRNA Expression Profiles in Male Gonads after siRNA Interference

#### 2.5.1. Overview of miRNAs in Gonads of Procambarus Clarkii

To gain an overview of miRNAs in gonads (AG and Te) of *P. clarkii* and identify miRNAs potentially involved in the regulation of *PcIAG* expression, eight small RNA libraries were constructed for the androgenic gland (AG) and testis (Te) tissues of four groups (control group and three siRNA injection groups) and sequenced with Illumina sequencing technology (Table S8).

All the clean reads were annotated based on the *P. clarkii* mRNA databases obtained in this study, and classified into nine categories including miRNA, rRNA, tRNA, snoRNA, snRNA, repbase, exons, introns, and those without any annotation. A total of 300 known miRNAs were identified and 374 new miRNAs were predicted (Table S9).

**Figure 3:**
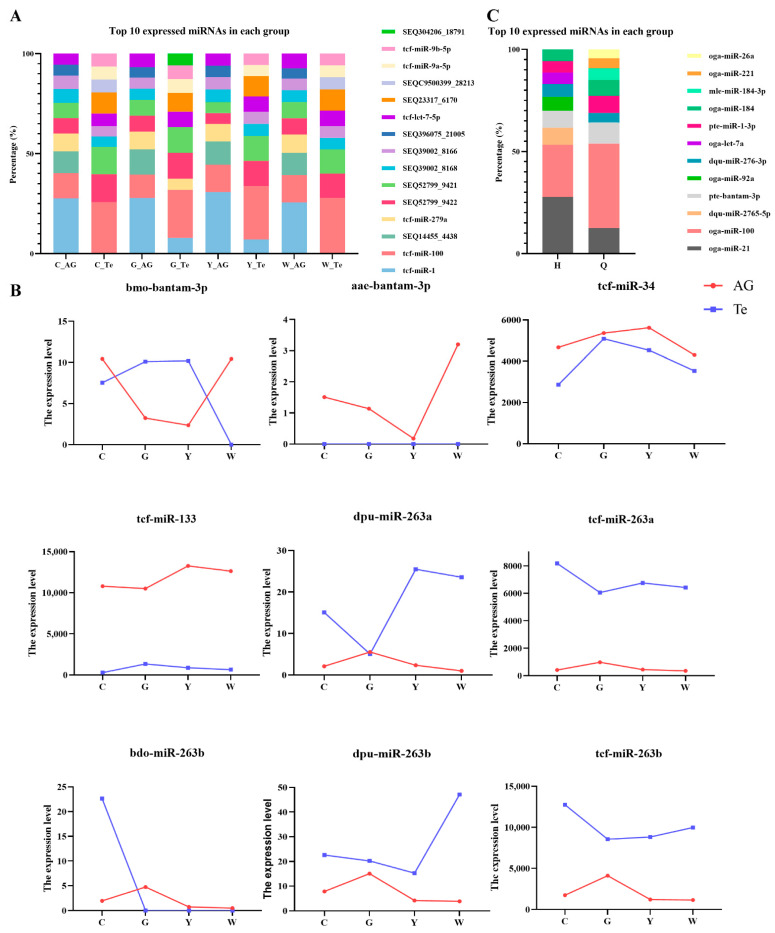
The expression of the top 10 miRNAs and sex-related miRNAs in miRNA expression profiles. (**A**) Distribution of top 10 miRNAs expression levels in samples. Te: Testis; AG: Androgenic gland. (**B**) Effects of siRNA interference on sex-related miRNAs. Te, testis; AG, androgenic gland. C denotes the *Procambarus clarkii* injected with saline (control group); G denotes the *Procambarus clarkii* injected with 0.02 µg/g body weight GsiRNA (treated group); Y denotes the *Procambarus clarkii* injected with 0.02 µg/g body weight YsiRNA (treated group); W denotes the *Procambarus clarkii* injected with 0.02 µg/g body weight WsiRNA (negative control). (**C**) Distribution of top 10 miRNAs expression levels in each group. Group Q, The exosomes in blood before androgenic gland ablation; Group H, The exosomes in blood after androgenic gland ablation.

The top 10 expressed miRNAs in gonadal tissues of four groups was shown in Figure 3A. In the GsiRNA and YsiRNA group, tcf-miR-1 expression increased in Te tissue compared to the control group, which became one of the top 10 expressed miRNAs in the GsiRNA and YsiRNA group. And SEQC9500399_28213 expression decreased in Te tissue compared to the control group. However, the types of the top 10 expressed miRNAs in Te tissues in the WsiRNA group did not change. The results indicated that tcf-miR-1 and SEQC9500399_28213 might play an important role in the regulation of *PcIAG* expression. In addition, in Te tissue, the expression of SEQ304206_18791 and tcf-miR-279a in the GsiRNA group increased compared to the control group and became one of the top 10 expressed miRNAs in the GsiRNA group. It indicated that there might be differences between the two siRNA (GsiRNA and YsiRNA) in regulating the expression of *PcIAG*.

#### 2.5.2. Differentially Expressed (DE) miRNAs

A total of 152 differentially expressed miRNAs were identified (Figure S8; Table S10). Based on the literature report [27,28], thirteen sex-related miRNAs were identified in this study, among which bantam-3p, miR-34, miR-133, miR-263a, and miR-263b were differentially expressed (Table 1). Among them, the expression changes of miR-133, miR-263a, and miR-263b were opposite after GsiRNA and YsiRNA injection (Figure 3B).

#### 2.5.3. Target Gene Prediction, GO Classification, and KEGG Pathway Analyses

In total, 1536 putative target genes for the 627 miRNAs were identified (Table S11). To better understand the regulatory roles of the differentially expressed miRNAs, GO enrichment and KEGG pathway analyses of the target genes of differentially expressed miRNAs were performed, and many GO terms and KEGG pathways were enriched (Table S12). The KEGG pathway analysis showed that target genes were enriched in the MAPK signaling pathway, which is a sex-related pathway [28]. The GO analysis indicated that the target genes were statistically enriched in more than 100 GO terms (Figure S9).

Target genes in the GsiRNA group vs. control group were enriched in four sex-related GO terms in Te tissue, including ovarian follicle cell stalk formation, female germ-line cyst encapsulation, cytoplasmic transport/nurse cell to the oocyte, ovarian follicle cell migration. The target genes in the YsiRNA group vs. control group were only enriched in negative regulation of canonical Wnt signaling pathway and regulation of canonical Wnt signaling pathway, while sex-related GO term was not enriched in the target genes in the WsiRNA group vs. control group (Table S13). The GO enrichment results of the target genes of differentially expressed miRNAs were similar to the above differentially expressed genes. However, there were also differences in the sex-related GO terms of the target genes of differentially expressed miRNAs between the GsiRNA group vs. control group and YsiRNA group vs. control group. It could be further confirmed that the small RNA interference pathway for regulation of *PcIAG* was different although there is only one base difference between the two siRNAs (GsiRNA and YsiRNA).

### 2.6. Analyses of Exosome miRNA

#### 2.6.1. Comparison of the Top 10 Highly Expressed miRNAs after siRNA Interference with miRNAs in Exosomes

The known miRNAs and new miRNAs identified in exosomes (Tables S14 and S15) were compared with the top 10 highly expressed miRNAs after siRNA interference, and three known miRNAs (miR-1, miR-100, and let-7-5p) and four new miRNAs (SEQ14455_4438, SEQ39002_8168, SEQ39002_8166, and SEQC9500399_28213) were identified (Table 2); The miR-1 and miR-100 were highly expressed in exosomes before and after androgenic gland ablation (Figure 3C). The let-7, a sex-related miRNA [29], and the top 10 miRNA expressed in Te and AG tissues after siRNA interference, increased after androgenic gland ablation (Figure 3A,C). Such results suggested that let-7 might play an important role in the sex regulation of *P. clarkii*.

#### 2.6.2. Differentially Expressed (DE) miRNAs

A total of eight miRNAs, including animal-mir-143-3; animal-mir-133-3; animal-mir-143-1; animal-mir-34-5; animal-mir-181-11; animal-mir-224-1; animal-mir-193-5; animal-mir-25-8, were differentially expressed in exosomes. Of the eight miRNAs, the four miRNAs (animal-mir-181-11; animal-mir-224-1; animal-mir-193-5; animal-mir-25-8) were upregulated, and the four miRNAs (animal-mir-143-3; animal-mir-133-3; animal-mir-143-1; animal-mir-34-5) were downregulated (Table S16). The eight differentially expressed miRNAs were mapped on either side of the volcano plot (Figure S10A). The hierarchical clustering of eight participants with the differentially expressed miRNAs revealed two distinct clusters (upregulated and downregulated expression of differentially expressed miRNAs) (Figure S10B). Among the eight differentially expressed miRNAs, the three differentially expressed miRNAs (miR-34, miR-133, miR-193) shown in Table 3 were also found in miRNA expression profiles after interfering with siRNA. Of which miR-133 [30] and miR-34 [28] were associated with sex.

The target genes of the eight differentially expressed miRNAs described above copredicted by Miranda software were 5506. GO enrichment analysis showed that the miRNA target genes were enriched in many sex-related GO terms, involving somatic sex determination, primary sex determination, female sex determination, germ-line sex determination, male sex determination et.al. In addition, KEGG pathway enrichment analysis showed that the miRNA target genes were enriched in the Wnt signaling pathway, GnRH signaling pathway, and MAPK signaling pathway, which were related to sex (Figure S10C,D; Table S17).

## 3. Discussion

Exploring the structure of *IAG* is an important basis for understanding the regulation of *IAG* expression and is of great significance to studying *IAG* expression regulation and function. In this study, the gene structure and complete promoter sequence of *PcIAG* were explored. It could provide a theoretical basis for understanding the sex differentiation and molecular regulation mechanism of *P. clarkii*. At present, the *IAG* DNA sequences of *M. nipponense*, *Scylla paramamosain*, *P. fallax,* and *F. chinensis* have been obtained [31,32,33,34]. The *IAG* DNA sequence of *P. clarkii* was comprised of four exons and three introns, similar to the *IAG* sequence of *P. fallax* and *S. Paramamosain*. Promoter core sequences TATA box and CAAT box were found in the 5′-flanking region of *M. rosenbergii IAG*, in addition, many potential transcription factor binding sites were predicted, such as SRY, Sox 5 and GATA 1 [35]. In this study, many potential transcription factor binding sites such as SRY, Sox2, and Sox13 were found in the 5′-flanking region of *PcIAG*. SRY and Sox were reported to be involved in male sex determination or differentiation [36,37]. Moreover, it was clear that there was no difference in the repeated sequences that male and female DNA contained, and the *IAG* cDNA sequence from AG tissue contained the completed repeated sequences region, whereas other tissues did not. However, there may be differences in the number of the two repeats, and the impact of such differences on the realization of *IAG* functions is required to be further studied.

Previous studies have shown that the 3′UTR seems to be important for the translation of *IAG*, and tandem repeats in the *IAG* 3′UTR of *M. rosenbergii* and *C. quadricarinatus* may regulate *IAG* translation [7]. Also, the 5′UTR plays an important role in gene expression regulation [38]. However, few studies of *IAG* 5′UTR are documented at present. In this study, two siRNAs were designed according to the two repeats in the 5′UTR, which successfully interfered with the expression of *PcIAG* in AG. In other tissues, GsiRNA and YsiRNA have a poor inhibitory effect or even fail to inhibit the expression of *PcIAG*. WsiRNA could not inhibit the expression of *PcIAG* in the five tissues. And the repeated sequences region of 5′UTR was found in the cDNA of AG, but not in the cDNA of other tissues. Different 5′UTRs and their contained elements show great differences in tissues and cells of different origins, that is, specific 5′UTRs can only exert optimal regulatory efficiency in specific tissues or cells [39]. It is suggested that the two repeats may be involved in the regulation of *PcIAG* expression in AG.

**Figure 4:**
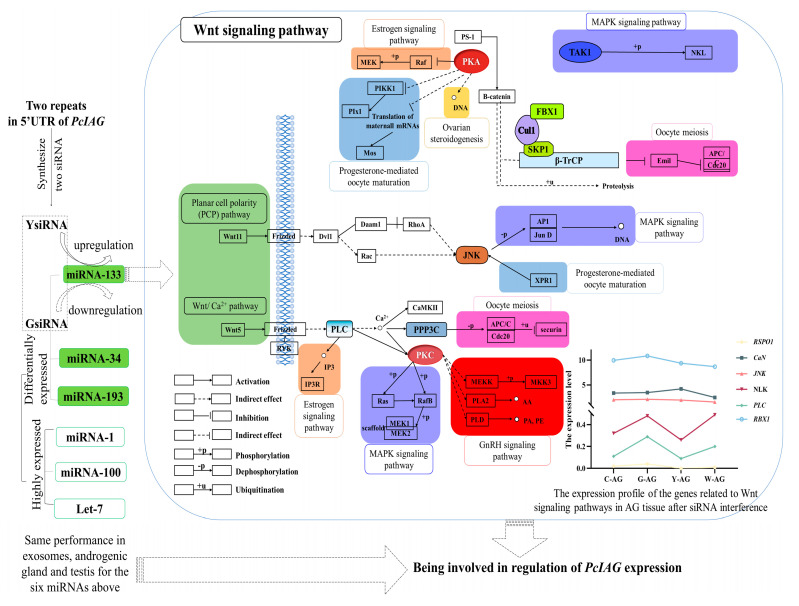
The miRNAs and Wnt signaling pathways being involved in *PcIAG* expression, the association of sex-related pathways with Wnt signaling pathways, and the expression profile of the genes related to Wnt signaling pathways after siRNA interference. Green-filled boxes denote the two pathways in the Wnt signaling pathway and miRNA involved in *PcIAG* expression. The pink-filled box denotes oocyte meiosis. The orange fill box denotes the estrogen signaling pathway. A blue-filled box denotes progesterone-mediated oocyte maturation. A yellow-filled box denotes ovarian steroidogenesis. The red-filled box denotes the GnRH signaling pathway. The purple-filled box denotes the MAPK signaling pathway.

In this study, the two siRNAs designed based on two repeats in the 5′UTR were used to interfere with *PcIAG* in *P. clarkii*. The mRNA and miRNA sequencing and analyses were performed to identify and analyze the sex-related differentially expressed genes, differentially expressed miRNAs, and the target genes of differentially expressed miRNAs. The results showed that sex-related GO terms were enriched in the target genes of the GsiRNA group vs. the control group and the YsiRNA group vs. the control group. However, although the WsiRNA group as a nontarget control increased the expression of *PcIAG* in each tissue due to some uncertain reason, the differentially expressed genes and the target genes of differentially expressed miRNAs from the WsiRNA group vs. the control group did not enrich to sex-related GO terms. It could be indicated that the two repeats had a close relationship with the regulation of *PcIAG* expression (Figure 4). The sex-related GO terms enriched by the differentially expressed genes in the GsiRNA group vs. control group and YsiRNA group vs. control group were completely different, indicating that although there was only one base difference between the two siRNA, the regulation of *PcIAG* may be different. The differentially expressed genes from mRNA sequencing were enriched in many sex-related pathways. The Wnt signaling pathway and other sex-related pathways can be linked through several genes (Figure 4), and the expression of these genes changed after siRNA interference. The target genes of the differentially expressed miRNAs in exosomes were also enriched in the pathways which related to the Wnt signaling pathway (Table S18). The Wnt signaling pathway plays an important role in the regulation of gonadal development and in the maintenance of undifferentiated spermatogonial cells [28,40]. The *Wnt4* gene was differentially expressed after siRNA interference. *Wnt4* played an important role in maintaining female morphogenesis [41]. The results indicated that *PcIAG* might play an important role in sex regulation, and the Wnt signaling pathway might be related to the regulation of *PcIAG*. The differentially expressed genes and the target genes of differentially expressed miRNAs in the YsiRNA group vs. the control group (but not in the GsiRNA group vs. the control group) were enriched in GO terms related to the Wnt signaling pathway. And the genes, including R-spondin 1 (*RSPO1*), Serine/Threonine-protein phosphatase 2B regulatory subunit (*CaN*), Mitogen-activated protein kinase (*JNK*), Nemo-like kinase (*NLK*), and Phosphatidylinositol phospholipase C (*PLC*), were related to Wnt signaling pathway, had opposite changes in AG tissues after GsiRNA and YsiRNA interference. It indicated that the difference in the regulation of *PcIAG* expression between GsiRNA and YsiRNA appeared to be related to the Wnt signaling pathway.

As an important transfer vehicle for intercellular communication and genetic material, exosomes can directly stimulate target cells through receptor-mediated interactions or through the transfer of various bioactive molecules such as lipids, proteins, mRNAs, and different non-coding RNAs and miRNAs, to perform their biological functions [42,43]. Valadi et al. [44] extracted miRNA and mRNA from exosomes and confirmed that exosomes secreted by mast cells could transport miRNA and mRNA and other substances to target cells. The expression levels of the miR-1, miR-100, and let-7 were highly expressed in exosomes, AG, and Te tissues (Figure 3A,C and Figure 4). Moreover, the expression levels of the three miRNAs were different before and after siRNA interference and androgenetic ablation. The differentially expressed miRNAs including miR-133, miR-193, and miR-34 were identified in exosomes, AG, and Te tissues (Table 3). Lou et al. [28] found that the expression of *IAG* in *Eriocheir sinensis* could be downregulated by miR-34 and let-7b. miR-1-3p as an intermediate male determiner is required for male sex determination in early embryogenesis in *Bactrocera dorsalis* [45]. The expression level of miRNA-100 was high in gonads of *E. sinensis* [30,46]. Song et al. [30] miR-133 exhibit differential expression during the meiotic maturation of the oocytes in *E. sinensis*. It indicated that the six miRNAs (miR-133, miR-193, miR-34, miR-1, miR-100, and let-7) above might be related to the regulation of *PcIAG* expression. The expression changes of miR-133 were opposite after GsiRNA and YsiRNA injection (Figure 3B and Figure 4). Moreover, miR-133 can be enriched to target genes related to the Wnt signaling pathway, such as *Wnt-5b*, indicating that the difference in the regulation of *PcIAG* by the two repeats may be related to miR-133.

## 4. Materials and Methods

### 4.1. Experimental Crayfish

The healthy *P. clarkii* were collected from the aquaculture base of Huazhong agricultural university. The crayfishes were reared in water tanks (water temperature, 24–26 °C; pH, 7–7.6; dissolved oxygen, 8–11 mg/L; ammonia nitrogen, 0–0.4 mg/L; nitrite, 0.005–0.01 mg/L). The crayfish were fed with a commercial pelleted feed (Tongwei Co., Ltd., Chengdu, China) at a dose of 2% of their body weight once a day until one week before the trial.

A total of 20 male adults (weighing approximately 20–35 g) were sampled for RNA-seq after RNAi with siRNAs. About 30 male adults (weighing approximately 25–40 g) had their blood collected for exosome sequencing after androgenic gland ablation. The muscle was collected from adult female and male *P. clarkii* and was used to amplify the *PcIAG* gene, its promoter, and the repeat regions. The muscle tissues were preserved in 95% ethanol for later DNA extraction. Samples from different tissues and developmental stages within the four-month life span of *P. clarkii* were collected for amplification of the repeat regions at the cDNA level. Different developmental stages including the NS, ZS, and the first to the 115th day after hatching. The embryos were sampled in NS and ZS, and the cephalothoraxes were sampled in larval stages. The tissues including AnGl, Mu, Gi, Es, Br, St, MG, Hep, PN, SG, OV, Te, and AG were collected from female and male *P. clarkii*. All the samples collected were immediately stored in liquid nitrogen.

### 4.2. Cloning and Analysis of PcIAG Gene

The modified proteinase K/ammonium acetate extraction protocol was used to extract DNA from the muscle of *P. clarkii* [47]. DNA was diluted to 100 ng/μL for further amplification. The primers (Table S19) for cloning *PcIAG* were designed based on the reference sequences KT343750.1 and PRJNA727411 in National Center for Biotechnology Information (NCBI, Version 2022. https://www.ncbi.nlm.nih.gov/ Accessed on: 20 June 2022). The *PcIAG* DNA fragments were assembled and obtained. The phylogenetic tree was constructed using the DNA sequences of *IAG* in crayfish, shrimp, and crab, which have been submitted, including *P. fallax* (MF405196.1), *P. virginalis* (MF405197.1), *S. paramamosain* (KJ870255.1), *M. nipponense* (KF811212.1) and *F. chinensis* (JQ388275.1). The phylogenetic tree was constructed using MEGA software version 5.0.

### 4.3. Cloning and Bioinformatics Analyses of PcIAG Promoter

The upstream 1363 bp fragment of *PcIAG* was obtained by amplification with the primers (Table S19) designed based on the genome database of *P. clarkii* (GenBank: PRJNA727411). The condition for amplification was as follows: 95 °C for 5 min, followed by 35 cycles of 95 °C for 30 s, 61.4 °C for 30 s, 72 °C for 1 min 30 s, and a final extension at 72 °C for 10 min. The amplification product was purified using the Gel Extraction Kit (Accurate Biotechnology, Hunan, China), cloned into pMD18-T Vector (Takara, Dalian, China), and transformed into Escherichia coli strain DH5α competent cells (Takara, Dalian, China) by following the manufacturer’s instruction. At least three clones were sent to Tsingke Biotechnology (Wuhan, China) for sequencing.

The promoter sequence and TSS of *PcIAG* were analyzed using the NNPP (Version 2022. https://www.fruitfly.org/seq_tools/promoter.html. Accessed on: 26 June 2022) and Promoter (Version v2.0. https://services.healthtech.dtu.dk/service.php?Promoter-2.0. Accessed on: 26 June 2022). Transcription factor binding sites (TFBS) were predicted by PROMO (Version 3.0.2. http://alggen.lsi.upc.es/cgi-bin/promo_v3/promo/promoinit.cgi?dirDB=TF_8.3. Accessed on: 26 June 2022). TATA-box was predicted by softberry (Version 2022. http://www.softberry.com/berry.phtml?topic=index&group=programs&subgroup=promoter. Accessed on: 26 June 2022). CpG island was predicted by CpGPlot (Version 2022. https://www.ebi.ac.uk/Tools/seqstats/emboss_cpgplot/. Accessed on: 26 June 2022) and Meth Primer (Version 2022. http://www.urogene.org/cgi-bin/methprimer/methprimer.cgi. Accessed on: 26 June 2022).

### 4.4. Amplification of PcIAG Repeat Regions in Male and Female Procambarus Clarkii DNA, and Different Tissues and Different Developmental Stages of Procambarus Clarkii

The total RNA from the collected tissues was extracted using a Trizol reagent (Invitrogen, CA, USA). Then, 1 µg of RNA was subjected to the reverse-transcription synthesis of cDNA using PrimeScriptTMRT reagent Kit with gDNA Eraser (TaKaRa, Dalian, China). Primers (*PcIAG*-F/*PcIAG*-R) were designed based on the full-length cDNA of *PcIAG* [48] to amplify fragments containing repeated sequences at DNA and cDNA levels (Table S19).

### 4.5. RNAi with siRNAs Designed Based on the Wwo Short Repeats in the 5′UTR of PcIAG

**Figure 5:**
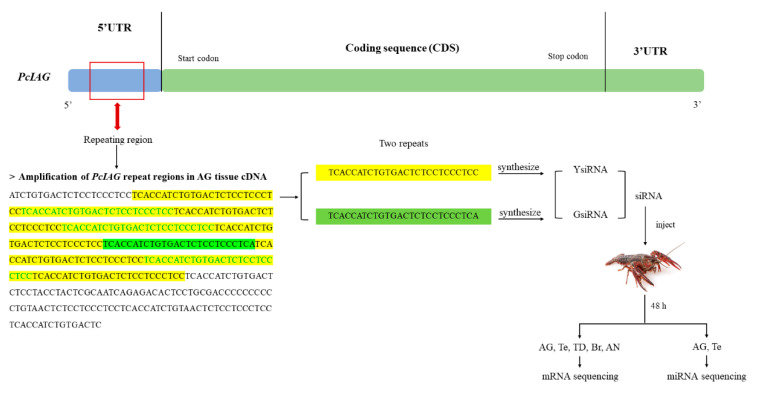
Experimental procedures for RNAi. Br: Brain; Te: Testis; AG: Androgenic gland; TD: Testicular ducts; AN: Abdominal nerve cord. GsiRNA denotes the *Procambarus clarkii* injected with 0.02 µg/g body weight GsiRNA (treated group); YsiRNA denotes the *Procambarus clarkii* injected with 0.02 µg/g body weight YsiRNA (treated group).

Two siRNAs were synthesized according to the two short repeats in the repeating region of 5′UTR, named GsiRNA and YsiRNA respectively (Figure 5). At the same time, WsiRNA was designed as a nontarget siRNA. For the assay, three adult male *P. clarkii* groups (n = 6 for each group) were injected with GsiRNA, YsiRNA, and WsiRNA, respectively, at a dose of 0.2 µg/g body weight. The other group was injected with saline as a control group. Te, TD, AG, Br, and AN were collected two days post siRNA injection and immediately preserved in liquid nitrogen. Each type of tissue was mixed and stored in liquid nitrogen for subsequent extraction of total RNA.

Total RNA was extracted by the Trizol method. The cDNA was reverse-transcribed with 1 µg of total RNA. Firstly, qPCR was used to determine the RNAi effect in the AG tissues. The specific primers *PcIAG*-qF/qR (Table S19) were designed to generate about 170 bp fragments. qPCR was performed using a QuantStudio™ Real-Time PCR System (Thermo Scientific, Waltham, MA, USA) in a 20-µL reaction mix, containing 2 µL of cDNA template, 0.2 µL of each primer (10 µM), 7.6 µL of water and 10 µL of TB GreenTM Premix Ex TaqTM (TaKaRa, Dalian, China). The qPCR program consisted of an initial denaturation at 95 ° C for 10 min, followed by 40 cycles of 95 °C for 30 s, 64 °C for 45 s, and 72 °C for 30 s. Three replicates were set. The *PcIAG* expression level was normalized to that of 18S-RNA (18S) and calculated using the 2^−∆∆Ct^ method and expressed as mean ± SD. Statistical analyses were performed using one-way ANOVA, and it was considered significant when *p* < 0.05 (*) or considered extremely significant when *p* < 0.01 (**). The charts were created using GraphPad Prism 8.0 (GraphPad Software, San Diego, CA, USA). Furtherly, the RNA samples prepared were sent to the Shanghai Majorbio Bio-pharm Biotechnology Co., Ltd. (Shanghai, China) for mRNA and miRNA sequencing. A total of 20 samples including all the gonadal and neural tissues were prepared for mRNA sequencing, and a total of eight samples including the testis and androgenic gland tissues were also collected for miRNA sequencing.

### 4.6. mRNA and miRNA Sequencing

For mRNA sequencing, RNA purification, reverse transcription, and library construction were performed according to the manufacturer’s instructions (Illumina, San Diego, CA, USA). The library was sequenced in a single lane on an Illumina NovaSeq 6000 sequencer (Illumina, San Diego, CA, USA) for 2 × 150 bp paired-end reads. The raw paired-end reads were trimmed and quality controlled by SeqPrep (Version 1.2. https://github.com/jstjohn/SeqPrep. Accessed on: 24 September 2019) and Sickle (https://github.com/najoshi/sickle) with default parameters. Then clean data from the samples (20 gonadal and neural tissues) were used to do de novo assembly with Trinity (Version v2.8.5. http://trinityrnaseq.sourceforge.net/. Accessed on: 24 September 2019). All the assembled transcripts were searched against the NR, COG, and KEGG databases using BLASTX to identify the proteins that had the highest sequence similarity with the given transcripts to retrieve their function annotations, and a typical cut-off E-values less than 1.0 × 10^−5^ was set. BLAST2GO (Version 2.5. http://www.blast2go.com/b2ghome. Accessed on: 24 September 2019) program was used to get GO annotations of unique assembled transcripts for describing biological processes, molecular functions, and cellular components. Metabolic pathway analysis was performed using the Kyoto Encyclopedia of Genes and Genomes (KEGG, Version 2018. http://www.genome.jp/kegg/. Accessed on: 24 September 2019). To identify differential expression genes between two different samples, the expression level of each transcript was calculated according to the transcripts per million reads (TPM) method. RSEM (Version 1.3.1. http://deweylab.biostat.wisc.edu/rsem/. Accessed on: 24 September 2019) was used to quantify gene abundances. Differential expression analysis was performed using the DESeq2 with Q value ≤ 0.05, DEGs with |log2FC| > 1. In addition, functional-enrichment analyses, including GO and KEGG, were performed to identify which differential expression genes were significantly enriched in GO terms and metabolic pathways at Bonferroni-corrected *p*-value ≤0.05 compared with the whole-transcriptome background. GO functional enrichment and KEGG pathway analysis were carried out by Goatools (Version 0.6.5. https://github.com/tanghaibao/Goatools. Accessed on: 24 September 2019) and KOBAS (Version 2.1.1. http://kobas.cbi.pku.edu.cn/home.do. Accessed on: 24 September 2019).

For miRNA sequencing, sequencing libraries were generated using NEBNext^®^ Multiplex Small RNA Library Prep Set for Illumina^®^ (New England Biolabs Inc.; Ipswich, MA, USA.) following the manufacturer’s recommendations. NEB 3′SR and 5′SR Adaptor were directly and specifically ligated to the 3′ and 5′ ends of miRNA, siRNA, and piRNA. The first strand of cDNA was synthesized using M-MuLV Reverse Transcriptase (RNase H–). PCR amplification was performed using LongAmp Taq 2X Master Mix, SR Primer for illumina and index (X) primer. PCR products were purified on 8% polyacrylamide gel (100 V, 80 min). DNA fragments corresponding to 140~160 bp (the length of small noncoding RNA plus the 3′ and 5′ adaptors) were recovered and dissolved in 8 μL elution buffer. Library quality was assessed on the Agilent Bioanalyzer 2100 system using DNA High Sensitivity Chips. The library preparations were sequenced on an Illumina NovaSeq 6000 sequencer (Illumina, San Diego, CA, USA) and 50 bp single-end reads were generated. The raw paired end reads were trimmed and quality controlled by SeqPrep (Version 1.2. https://github.com/jstjohn/SeqPrep. Accessed on: 20 November 2019) and Sickle (Version 1. https://github.com/najoshi/sickle. Accessed on: 20 November 2019) with default parameters. Then clean reads were separately aligned to reference genome with orientation mode using HIASAT (https://ccb.jhu.edu/software/hisat2/index.shtml) software. The mapped reads of each sample were assembled by StringTie (Version 1.3.3b. https://ccb.jhu.edu/software/stringtie/index.shtml?t=example. Accessed on: 20 November 2019). Low-quality bases (Sanger base quality of <20) of the 3′ ends were trimmed, and then the sequencing adapters were removed with the fastx toolkit software (Version 0.0.14. http://hannonlab.cshl.edu/fastx_toolkit/. Accessed on: 20 November 2019). All identical sequences of sizes ranging from 18 to 32 nt were counted and eliminated from the initial data set. The assembled unique sequences were used for a BLAST search of the Rfam database (Version 10.1 http://rfam.sanger.ac.uk/. Accessed on: 20 November 2019), to remove non-miRNA sequences (rRNA, tRNA, snoRNA, etc.). Bowtie (http://bowtie-bio.sourceforge.net/index.shtml) was used to annotate the chromosomal location against the reference genome data. Through a BLAST search of the miRbase (Version 21.0. http://www.mirbase.org/. Accessed on: 20 November 2019), the perfectly matched sequences were used to count and analyze the known miRNA expression profile. The characteristics of the hairpin structure of miRNA precursor can be used to predict novel miRNA. The available software mireap was to predict novel miRNA, the Dicer cleavage site, and the minimum free energy of the small RNA tags unannotated in the former steps. The expression level of each miRNA was calculated according to the transcripts per million reads (TPM) method. Significant differently expressed miRNAs were extracted with |log2FC| > 1 and FDR < 0.05 by DEseq2. In order to illustrate the biological functions of the miRNAs, the potential target genes of these miRNAs were predicted using Miranda software. The putative targets were annotated by the Goatools (Version 0.6.5. https://github.com/tanghaibao/Goatools. Accessed on: 20 November 2019), and the KEGG pathway analysis was carried out by KOBAS (Version 2.1.1. http://kobas.cbi.pku.edu.cn/home.do. Accessed on: 20 November 2019).

The raw data of mRNA and miRNA after GsiRNA, WsiRNA, and saline injection could be obtained under accession PRJNA823494 (mRNA) and PRJNA824877 (miRNA). The raw data of mRNA and miRNA after YsiRNA injection were submitted to the NCBI Sequence Read Archive (SRA) database with under accession PRJNA823571 (mRNA) and PRJNA824954 (miRNA).

### 4.7. Exosome miRNA Sequencing

Androgenic gland ablation was performed by the method described by Barki et al. [4]. The blood samples were collected from the crayfish before and 2–3 days after androgenic gland ablation (when the wound was almost healed). The blood (about 200–500 μL) was extracted from each crayfish with a 1 mL disposable sterile syringe and was pooled with an equal volume of ACD anticoagulant (Citric acid monohydrate, C6H8O7·H_2_O, 0.48 g; Trisodium citrate dihydrate, C6H5Na3O7·_2_H_2_O, 1.32 g; D-(+)-glucose, C6H12O6·H_2_O, 1.47 g; ddH_2_O 100 mL). The blood from the 30 individuals was mixed, and then centrifuged at 1200 r/min at 4 °C for 10 min in a refrigerated centrifuge, and the upper plasma was removed into a 1.5 mL centrifuge tube. The plasma was then centrifuged at 13,000 r/min at 4 °C for 2 min in a refrigerated centrifuge, and the supernatant was removed into a frozen pipe quick-frozen with liquid nitrogen for 1 h, stored at −80 °C for further experiments. Exosome extraction and sequencing were carried out at the Shanghai Personal Biotechnology Co., Ltd. (Shanghai, China).

Sequencing libraries were generated using NEB Next^®^ Multiplex Small RNA Library Prep Set for Illumina^®^ (New England Biolabs Inc.; Ipswich, MA, USA). Library quality checks were performed using Agilent 2100 Bioanalyzer (Agilent Technologies Inc., Santa Clara, CA, USA) and Agilent High Sensitivity DNA Kit (Agilent Technologies Inc., Santa Clara, CA, USA, 5067-4626). The total library concentration was detected by Pico green (Quantifluor-ST fluorometer, Promega, Madison, WI, USA, E6090; Quant-iT PicoGreen dsDNA Assay Kit, Invitrogen, California, USA, P7589), and the effective library concentration was quantified by qPCR (StepOnePlus Real-Time PCR Systems, Thermo Scientific, Waltham, MA, USA). The libraries were sequenced in PE150 mode on an Illumina sequencer.

All identical sequences of sizes ranging from 18 to 36 nt were counted and eliminated from the initial data set. Then reads were separately aligned to the *P. clarkii* reference genome (GenBank: PRJNA727411) using miRDeep2 (Mackowiak SD, 2011) software, in which the mapper.pl program called Bowtie to align Unique Reads with the reference genome sequence.

The precursor and mature sequences of miRNAs of *P. clarkii* were downloaded from miRBase, and then the deduplicated sequences were aligned with them respectively to annotate the detected miRNAs. According to the alignment results with the genome, using mireap (v2.0) to analyze the sequences that have not been annotated with any information, and perform a new miRNA prediction analysis. Differential expression analysis between the two comparison combinations was performed using DESeq software (Version 1.30.0.). Significant differently expressed (DE) miRNAs were extracted with |log2FC| > 1 and FDR < 0.05 by DEseq. For miRNA target prediction Using Miranda, the 3‘UTR’ sequence of the mRNA of *P. clarkii* was used as the target sequence to predict the target gene of the differentially expressed miRNAs sequence. The putative targets were annotated by the topGO enrichment analysis, calculate the *p*-value by the hypergeometric distribution method (the standard of significant enrichment is *p*-value < 0.05), and find out the GO terms with significant enrichment of target genes. The KEGG pathway enrichment analysis of target genes of differentially expressed miRNAs was deduced using clusterProfiler (Version 3.4.4.), focusing on significantly enriched pathways with a *p*-value < 0.05.

## 5. Conclusions

The repeated sequences region of 5′UTR was found in the cDNA of AG, but not in the cDNA of other tissues, and the sequences were similar in male and female DNA. After interfering with siRNA, sex-related genes such as *Vg*, *Dmrt*, *Wnt4,* and *Sxl* were identified. The differentially expressed genes and the target genes of differentially expressed miRNAs were enriched in sex-related pathways such as the Wnt signaling pathway, Oocyte meiosis, Estrogen signaling pathway, and GnRH signaling pathway, indicating the importance of *IAG* in sex regulation. The differentially expressed genes were enriched in sex-related GO terms after the GsiRNA and YsiRNA interference, while were not enriched in sex-related GO terms after WsiRNA (as a nontarget one) interference. It indicated that the two repeats might be related to the regulation of *PcIAG* expression in AG tissue. The difference in the GO enrichment results of differentially expressed genes and the target genes of differentially expressed miRNAs of the two siRNA groups indicated that although the two repeats differ by only one base, they were different in regulating the expression of *PcIAG*. And the difference in regulation appears to be related to the Wnt signaling pathway. Moreover, six miRNAs, miR-133, miR-193, miR-34, miR-1, miR-100, and let-7 were identified in exosomes, and the miRNA sequencing results of gonadal tissue after interference, which may be involved in the regulation of *PcIAG* expression. Of the six miRNAs, miR-133 may be related to the regulation of *PcIAG* expression by these two repeats. The results of this study illustrated that *PcIAG* played important roles in sex regulation, and the two repeats were related to the regulation of *PcIAG* expression, which could enrich the molecular regulation of IAG in *P. clarkii* and provide references for the sex-related study in *P. clarkii*.

## Figures and Tables

**Table 1:** Sex-related miRNA in male gonads (AG, Te) of *Procambarus clarkii*.

	miRNA	Sequence	Whether Differential Expression
bantam-3p	aae-bantam-3p	UGAGAUCAUUUUGAAAGCUGAU	yes
	bmo-bantam-3p	UGAGAUCAUUGUGAAAGCUAAUU	yes
Let-7	aae-let-7	UGAGGUAGUUGGUUGUAUAGU	
	bdo-let-7	UGAGGUAGUAGGUUGUAUAGU	
miR-7	dpu-miR-7	UGGAAGACUAGUGAUUUUGUUGU	
	tcf-miR-7	UGGAAGACUAGUGAUUUUGUUGUU	
miR-9a	aae-miR-9a	UCUUUGGUUAUCUAGCUGUAUGA	
miR-9a-5p	bmo-miR-9a-5p	UCUUUGGUUAUCUAGCUGUAUGA	
	dme-miR-9a-5p	UCUUUGGUUAUCUAGCUGUAUGA	
	tcf-miR-9a-5p	UCUUUGGUUAUCUAGCUGUAUGA	
miR-10	dpu-miR-10	UACCCUGUAGAUCCGAAUUUGU	
miR-34	dpu-miR-34	UGGCAGUGUGGUUAGCUGGUUGUG	
	tcf-miR-34	UGGCAGUGUGGUUAGCUGGUUG	yes
miR-133	aae-miR-133	UUGGUCCCCUUCAACCAGCUGU	
	dpu-miR-133	UUGGUCCCCUUCAACCAGCUGU	
	tcf-miR-133	UUGGUCCCCUUCAACCAGCUGU	yes
miR-190	aae-miR-190	AGAUAUGUUUGAUAUUCUUGGUUG	
	bdo-miR-190	AGAUAUGUUUGAUAUUCUUGGUUG	
	tcf-miR-190	AGAUAUGUUUGAUAUUCUUGGUUG	
miR-190-5p	bmo-miR-190-5p	AGAUAUGUUUGAUAUUCUUGGUU	
miR-263a	dpu-miR-263a	AAUGGCACUGGAAGAAUUCAC	
	tcf-miR-263a	AAUGGCACUGGAAGAAUUCACGGG	yes
	bdo-miR-263a	AAUGGCACUGGAAGAAUUCACGGG	
miR-263b	bdo-miR-263b	CUUGGCACUGGGAGAAUUCACAG	yes
	tcf-miR-263b	CUUGGCACUGGAAGAAUUCACAGA	yes
	dpu-miR-263b	CUUGGCACUGGAAGAAUUCACA	yes
miR-307	dpu-miR-307	UCACAACCUCCUUGAGUGAG	
	tcf-miR-307	UCACAACCUCCUUGAGUGAGUG	

**Table 2:** Comparison of the top 10 highly expressed miRNAs after siRNA interference with miRNAs in exosomes before and after androgenic gland ablation.

After siRNA Interference with *PcIAG*, the Top 10 miRNAs Expressed	miRNA Sequence	miRNAs in Exosomes before and after the Androgenic Gland Ablation
tcf-miR-1	UGGAAUGUAAAGAAGUAUGGAG	pte-miR-1-3p
tcf-miR-100	AACCCGUAGAUCCGAACUUGUGU	hpo-miR-100-5p
tcf-let-7-5p	UGAGGUAGUAGGUUGUAUGGUU	oga-let-7c
tcf-miR-9a-5p	UCUUUGGUUAUCUAGCUGUAUGA	
tcf-miR-9b-5p	UCUUUGGUGGUCUAGCUGUAUGA	
tcf-miR-279a	UGACUAGAUCCACACUCATCCA	
SEQ14455_4438	UUGAGCAAAGCUUCAGGGGGUUU	animal-m0537-3p
SEQ23317_6170	AAAUAUCAGCUGGUAAAUUUGG	
SEQ52799_9422	UAUUAUGCUAAGAUUCGUGUAU	
SEQ52799_9421		
SEQ39002_8168	CAUCACAGUGAUAGUACCUUACU	animal-m0495-3p
SEQ39002_8166		
SEQ396075_21005	CCAAAAGGCCGAGAAGCGAUCACAU	
SEQ304206_18791	CCCUCAGGAUAGCUGGAAC	
SEQC9500399_28213	UGACUAGAGGACUACUCAUCC	animal-m0377-3p

**Table 3:** The same differentially expressed miRNAs in male gonads (AG, Te) and exosomes of *Procambarus clarkii*.

Differentially Expressed miRNAs after siRNA Interference	miRNA Sequence	Differentially Expressed miRNA in Exosomes	miRNA Sequence
tcf-miR-133	UUGGUCCCCUUCAACCAGCUGU	animal-mir-133-3	UUGGUCCCCUUCAACCAGCUGU
aae-miR-193	UACUGGCCUACUAAGUCCCAAC	animal-mir-193-5	AACUGGCCCUCAAAGUCCCGCU
tcf-miR-193	UACUGGCCUGCUAAGUCCCAAG		
tcf-miR-34	UGGCAGUGUGGUUAGCUGGUUG	animal-mir-34-5	AGGCAGUGUAGUUAGCUGAUUGC

## Data Availability

All sequenced samples have been submitted to the NCBI Sequence Read Archive Centre (SRA) with accession bioproject number PRJNA823494 (mRNA after GsiRNA, WsiRNA, and saline injection), PRJNA824877 (miRNA after GsiRNA, WsiRNA, and saline injection), PRJNA823571 (mRNA after YsiRNA injection) and PRJNA824954 (miRNA after YsiRNA injection).

## Supplementary Materials

The following supporting information can be downloaded at: https://www.mdpi.com/article/10.3390/ijms231810348/s1.
