# Supplementary material for: Two Short Repeats in the 5′ Untranslated Region of Insulin-like Androgenic Gland Factor in Procambarus clarkii (PcIAG) That Regulate PcIAG Expression
Source: Int J Mol Sci. 2022 Sep 7;23(18):10348. doi: 10.3390/ijms231810348 (PMC9499548; doi:10.3390/ijms231810348)
Supplement: Supplementary file 1 [file ijms-23-10348-s001.zip › Supplementary Materials Figure.pdf]

ACTATTCACGTCCCACTCACGCTGTATTCCTCCTGTAATACAAGACACTGGATACAAGCGATTCCTCTT
GCTGTAACTCCACGATGCTCACTCAAACATTACTGAAACTGN…5326bp…NGAAGGTAGTTGTGCTTTC
TGGGTTCGAGTCCCACTGGTGGGTGTTGTCCAAAGATTGTTTATCTTCACTTGTGGTTTATGCAAGTAT
AGGCTTATAAGCTGGACACGAGTTCTCTCACATTGACAGTGGCTTGACGAAAATTGCAGACTGACCCC
TCACTCATCTGGTGCCTTCGGGAGGTAGAGATGTTTGAGATATATATATCTCGAACTATCTACTTCTTTT
GTAAGGTCTGATGGCGTAGTGGGTTAAAGCATACTAGTTATGCCAGCTACTGGAAGGTAGTTGTGCTTT
CTGGGTTCGAGTCCCACTGGTGGGTGTTGTCCAAAGATTGTTTATCTTCACTTGTGGTTTATGCAAGTA
TAGGCTTATAAGCTGGACACGAGTTCTCTCACATTGACAGTGGCTTGACGAAAATTGCAGACTGACCC
CTCACTCATCTGGTGCCTTCGGGAGGTAGAGATGTTTGAGATATATATATCTCGAACTATCTACTTCTTTT
GTAAGGTCTGATGGCGTAGTGGGTTTAAAGCATACTAGTTATGCCAGCTACTGGAAGGTAGTTGTGCTT
TCTGGGTTCGAGTCCCACTGGTGGGTGTTGTCCAAAGATTGTTTATCTTCACTTGTGGTTTATGCAAGT
ATAGGCTTATAAGCTGGACACGAGTTCTCTCACATTGACAGTGGCTTGACGAAAATTGCAGACAAACA
ATCTTTGGACCTTGTCCAAAGATTGTTTATCTTCACTTGTGGTTTATGCAAGTATAGGCTTATAAGCTGG
ACACGAGTTCTCTCACATTGACAGTGGCTTGACGAAAATTGCAGACTGACCCCTCACTCATCTGGTGC
CTTCGGGAGGTAGAGATGTTTGAGATATATATATCTCGAACTATCTACTTCTTTTGTAAGGTCTGATGGC
GTAGTGGGTTAAAGCATACTAGTTATGCCAGCTACTGGAAGGTAGTTGTGCTTTCTGGGTTCGAGTCCC
ACTGGTGGGTGTTGTCCAAAGATTGTTTATCTTCACTTGTGGTTTATGCAAGTATAGGCTTATAAGCTGG
ACACGAGTTCTCTCACATTGACAGTGGCTTGACGAAAATGCAGACTGACN…11088bp…NAAATAAAG
AAAATTATTGAGATACATAAAATTAGAAAAATGAGATTTCCACATACTAAATATAATAATTACTTAATAAT
GTTAGGTACCCAGCTAAGGTATTCTTACTACATGACCACCTATTTTATAGATGACACTAATAGTAAAGGA
TATAACCTGTTAAATTAAAAATAATATAAATAGAATGATTACAGGTAAATGTTGACATTAAAATATGTACA
GTACTTCAATACATAAATACTGGCCTAGGCGAGGTTAGGTAAGGAGTTATTCTTTATTATTTTTAATTTTC
CACGTTATATGAGTAAGAGATTTTAAAGTATAAACGGTATTTGAATGCAGCAAATATTAAAAAACTTAGT
TTTTCTTTCCTAATTAGAGTTGGAACATTGGATTAGTCTTCAGTATTTAATGTGCCATGAATAAGTTAATT
AATTTGGAAGCTTCTAATTACTAAAAATACTTCATAACTTTAATTATGGGTGAATTATTTCTATTAACAAA
ATATTTGGTATGGAAAAGCACGACTTATCTTACTTTTTTATTTATTTAATTATTTATATATTCAAGATTTCTT
ACAATCTTATAAAGCCACTAGCACGCATAGCATTTCGGGCAGGTCCTAAATCCCAATTTTCCCCGAATA
CGACCCGCCAAATAGTTTAACAACAGGTACCCATTCACTGCTGGATGAACAGAGGCTCGTTAAGGATT
GGCGGCCTGTCAATCCTCCCTGGCCAGGATACGAACCCAGGCCAAAGCGCCGGGCGAATGTCTAAGA
CGAAAGTATAAATGAAAACGCAGAAGTTTAAAAGAATAGGAGTTAGTATTGCCTTATTAAGGATTAATA
TAATAATTTTCTGTCTTTTCATAAAAGACATATTTAATACGAATTACAGACACACAAAGCATATATTACTG
GCAGGACGCGATTATTATCTGCCGGCTAAGGTATTTTGAGTCCGGCTTGTGTTGTCGAGAAAACTTGAC
TAATGAACGGTAAAAGGTTTACGTAAAGTTACGACTGCAGTGAGGGTGN…431bp…NATACTGCAGGT
GCTGGTGGCGGTGTTGCGGTCGCTGCCATCCTCCTCGTACTGGGTAGAAAACCTTCTGGTGGACTTCG
ACTGCGGTAACCTGGCGGACACGATGGACAGTATATGTCTCACCTTCAACGAATACAACGATACTCATC
TGCACTATGCGGCCAGAGGTGCGTACGTGTGTGTGCTCACATTGGAATGTTTGTAAGGGTTGGGTTTTA
GCTCTAACATACAAACATCCAGGTATAGCACTTACTAGTTTGTCCCTTCAGCGGTGAGATCGGCGAGTG
GTGAGGCTCCAGCAGCCACTGTGACGATCCTGGACCCCAGAAGCCATGCCGGGCTTCCTCATGAACA
AGCCACACACCTGTCCGGGTTCGATCAGTTGTACCATGCACAAGTCAGGCACCTGGCCGGGCTTTCTC
AGCTGTACCATGCACAAGCTAGACACCACCCCGCTGAGGACATCACTCTTGTTGACCAAGTGAGTACA
ATTATCACCGTTGCTATCTCTCAACATTAATGCAAATTACTTAACTGAAAGAAAAATAGACTTTAGAGTT
TGAACCCATTATGCGACCAACTTTTATGCCTCCGCCCACAAGATGGGTATTGAATTCATAATAAACTAGT
TAAACTTTGTCGCAACCAGTTAATGAAGATGAAATAGTATAAATAATTATTAGGCTATCAGATTACTGTT
GTATACTTCAATATTTATTCAAGGAAGTATACAAGCACACTTCCAAACTTCGCCCTTCATACTCTTAAAT

TTTTGTGACATCAAATCATGAAAAACTTATGTTCGCAAATTCCATCTATAGGCAAAGAAGTATACAACG
AAGATATGATAGCCTCATGATTAATATTCTTTATGTCATCACGGCTTAGCGCTTGTGATGTCAACACTGC
CACAGACAGTGTTGACATCCGACTCACTGATGTAAACACTGTCACAGACAGTGTTGCCATCCGACTCA
CTGATGTAAACACTGTCACAGACAGTGTTGCCATCCGACTCACTGATGTAAACACTGCCACAGACCGA
TTCACTGATGTCAACACTACCACAGACCGACTCACTGACCTCAACACTACCACAGACCGACTCACTGA
TCTCAACACTGCCACAGACCGACTCACTGATCTCAACACTACCACAGACCGACTCACTGACCTCAAC
ACTACCACAGACCGACTCACTGATCTCAACACTGCCACAGACCGACTCACTGATCTCAACACTACGAC
AGACCGACTTACTGATGTCAATACTACCACAGACCGACTCACTGATCTCAACACTACCACAGACCGAC
TTACTGATGTCAACACTACCACAGACCGACTCACTGATCTCAACACTACCACAGACCGACACCCATCG
TCAGGACGTTGCTCTGTACATAACTCGACAGATAAGTTATTATTTTACTTCTGCACAAACATCAGCATAT
TTTAGTTACATGCCGAAATCAAGAATTAAACGACATTGTCCCGTTTTTACTTTAATATTTGCAGGTTAGC
ACTGATGAGGATCGGAAGATGGCGTTGCTGAGCCGCCAGGCCGCTCACACCTTCGTCAAGACACAGA
CCCGCCGCCATCGCCGTCAAGCCAACACAGACAACCATGTTCGCTTCAACATTCAGGACGAGTGTTGC
AATTATATGAGACCCAGGACCTGCGTCCTGGAGGAGATCACAGAGTACTGTGTCGAGCCGGAGGACG
GCGCCTTGCTCACCTGGTGAGGTGAGGGAGCCTCCAGAAGCCATACTACCCGGCCCAAGGGGGGTAG
AAATAGCCTAAGCTACTCTATCCCTTTGAGATGTATATTTTCTTGTCTCAATAAACATACTTGAACTTGA

Figure S1 The DNA sequences of *PcIAG*. The exon1 (exon1 = 110 bp) was marked with green color, the exon2 (exon2 = 116 bp) was marked with bule color, the exon3 (exon3 = 212 bp) was marked with yellow color, the exon4 (exon2 = 345 bp) was marked with purple color (GenBank: PRJNA727411). The red and blue fonts noted the two CpG islands were predicted by CpGPlot and CpG islands. Intron1 = 18992bp; Intron 2 = 103bp; Intron 3 = 1034 b

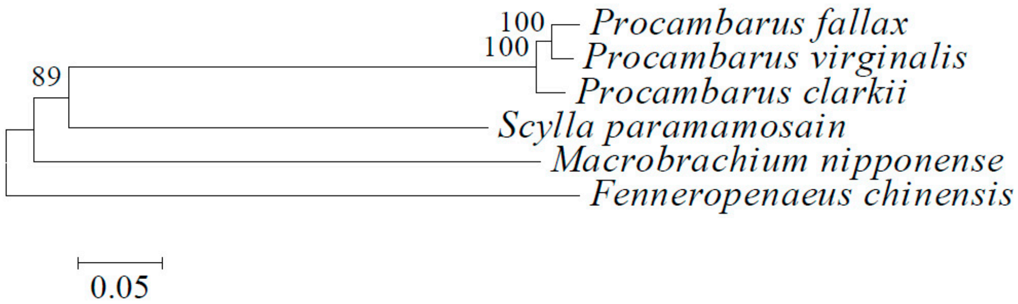

Figure S2 Phylogenetic tree of *PcIAG*. The phylogenetic tree was inferred using the neighbor-joining method. The values at the tree nodes are neighbor-joining bootstrap values. The DNA sequences used for phylogenetic analysis were as follows: *Procambarus fallax* (MF405196.1), *Procambarus virginalis* (MF405197.1), *Scylla paramamosain* (KJ870255.1), *Macrobrachium nipponense* (KF811212.1) and *Fenneropenaeus chinensis* (JQ388275.1).

CGTGTGAGGTGCAAACTATGAAATTATCTGTGCGTTATATAAGTAGTTGAACATCCTATTTCTTATTAAA -701

CCAATTCCTACATAAATATGATATGAACCATAATTATACGCCCACGGAACCATCTACCCGCCGAAGCCGT -631

AAATAGTAACATATTAATTCAGTTTAAAATTAAAATTCAGCCGGAAAATATCCTCAGGAATAATTGAGAG -561

ACCTTTGACAAGTCGCCGGCTTCCTGACCCCGTCAGGTTGACTAGAAATGGCGGCTCCTCAGGTCAAT -491

AAGAACATAAGGATAGAGGAAACTGCAGAAGGCCTATTGACCCATACGAAACATCTAGTTACATCCAC -423

CCAAACTCATTCATGTATCCAGTCATGCGACATCTATTATATTTCCCGCAAATTTGTTCTATAAATCAACA -355

ACCCTCTTTCCAAACCAGTATTTACCCAGGACTTTCCTGAATCTAATCTTATCTAATATATATATATATATC -284

CATGTAAATTAAATTTTAGGGACACCCCCAAAATCACACATCGGCTGACAACAGATTCCATTCTTGACT -212

CACTGGCATCAGCAGTTACCTCCCCCCCCCACCCCCACACCCTCTTCCGTATTAAAGGTGGTATGCCT -143

GTGACAATGGTGTGGTGCAGTGTGGTGACGTCACAGGTAGGGCTATATAGCTGCAGGTGTGGCCTCAG -74

TGGAGC**A**GGTGTGGCACCAGCAGCGGCCCTCTGCACTCCTCCGGCGGCACTCCCCTGTCCCAACTGG +60

TSS

CGGCCCCCTCACCATCTGTGACTCTCCTCCCTCCTCACCATCTGTGACTCTCCTCCCTCCTCACCATCTG +130

TGACTCTCCTCCCTCATCACCATCTGTGACTCTCCTCCCTCCTCACCATCTGTGACTCTCCTCCCTCCTC +200

ACCATCTGTGACTCTCCTCCCTCCTCACCATCTGTGACTCTCCTCCCTCATCACCATCTGTGACTCTCCT +270

CCCTCCTCACCATCTGTGACTCTCCTCCCTCATCACCATCTGTGACTCTCCTCCCTCCTCACCATCTGTG +340

ACTCTCCTCCCTCCTCACCATCTGTGACTCTCCTCCCTCCTCACCATCTGTGACTCTCCTCCCTCCTCAC +410

CATCTGTGACTCTCCTCCCTCCTCACCATCTGTGACTCTCCTCCCTCCTCACCATCTGTTGCTCCACTAC +480

AGACAGGGAGCCACAGACACGCGTGTGACCCCCCCCCAAAAAAGTGATACAGTACCGCTAGCTTAT +547

AAACCACTATTCACGTCCCACTCACGCTGTATTCCTCCTGTAATACAAGACACAGGATACAAGCGATTC +616

CTCTTGCTGTAACTCCACGATGCTCACTCAAACATTACTGAAACT +661

TIS

Figure S3 The promoter sequences of *PcIAG*. The two repeats in the repeating region were marked with green color and yellow color, respectively. Core promoter was shown in blue font and the TATA box was circled in red. TSS showed the transcriptional start site and TIS showed the translation start site of *PcIAG*.

>Female

TCTTTGATCTCCTCCTCCTCACCATCTGTGACTCTCCTCCCTCCTCACCATCTGTGACTCTCCTCCC

TCATCACCATCTGTGACTCTCCTCCCTCCTCACCATCTGTGACTCTCCTCCCTCCTCACCATCTGTG

ACTCTCCTCCCTCCTCACCATCTGTGACTCTCCTCCCTCATCACCATCTGTGACTCTCCTCCCTCCT

CACCATCTGTGACTCTCCTCCCTCATCACCATCTGTGACTCTCCTCCCTCCTCACCATCTGTGACTC

TCCTCCCTCCTCACCATCTGTGACTCTCCTCCCTCCTCACCATCTGTGACTCTCCTCCCTCCTCACC

ATCTGTGACTCTCCTCCCTCCTCACCATCTGTGACTCTCCTCCCTCCTCACCATCTGTTGCTCCACT

ACAGACAGGGAGCCACAGACACGCGTGTGACCCCCCCCCCAAAAAAAGGGATACAGTATCGCT

ACTTTATAAACCATTATTCACGTCCCACTCACGCTGAATTCCTCCGGTAATACAAAACCCGGGATA

CAAGCAATTCCTCTTGCTGAAACTCCACAATGCTCACTCAAACATTTATTAAAA

>Male

TCTATGATCTCCTCCCTCCTCACCATCTGTGACTCTCCTCCCTCCTCACCATCTGTGACTCTCCTCC

CTCATCACCATCTGTGACTCTCCTCCCTCCTCACCATCTGTGACTCTCCTCCCTCCTCACCATCTGT

GACTCTCCTCCCTCCTCACCATCTGTGACTCTCCTCCCTCATCACCATCTGTGACTCTCCTCCCTCC

TCACCATCTGTGACTCTCCTCCCTCATCACCATCTGTGACTCTCCTCCCTCCTCACCATCTGTGACT

CTCCTCCCTCCTCACCATCTGTGACTCTCCTCCCTCCTCACCATCTGTGACTCTCCTCCCTCCTCAC

CATCTGTGACTCTCCTCCCTCCTCACCATCTGTGACTCTCCTCCCTCCTCACCATCTGTTGCTCCAC

TACAGACAGGGAGCCACAGACACGCGTGTGACCCCCCCCCCCAAAAAAGGGATACAGAATCGT

TAGTTTATAAACCACAATTCACGTCCCATTCACGCTGAATTCCTCCGGTAATACAAAACCCGGGAT

ACAAGCAATTCCTCTTGTTGAAACTCCACAATGCTCACTCAAACTTTTATTAAAA

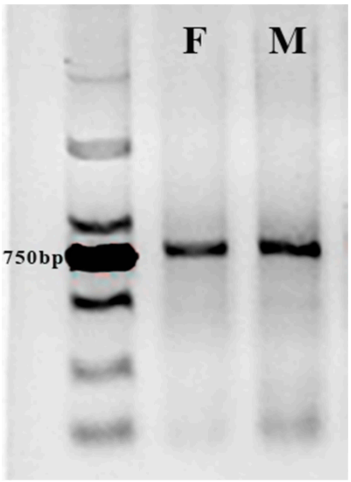

Figure S4 Repeat sequences in the DNA of female and male *Procambarus clarkii*. The translation start site was marked in red. The two repeating sequences of the repeating region were marked with green color and yellow color, respectively. F denotes female crayfish, M denotes male crayfish.

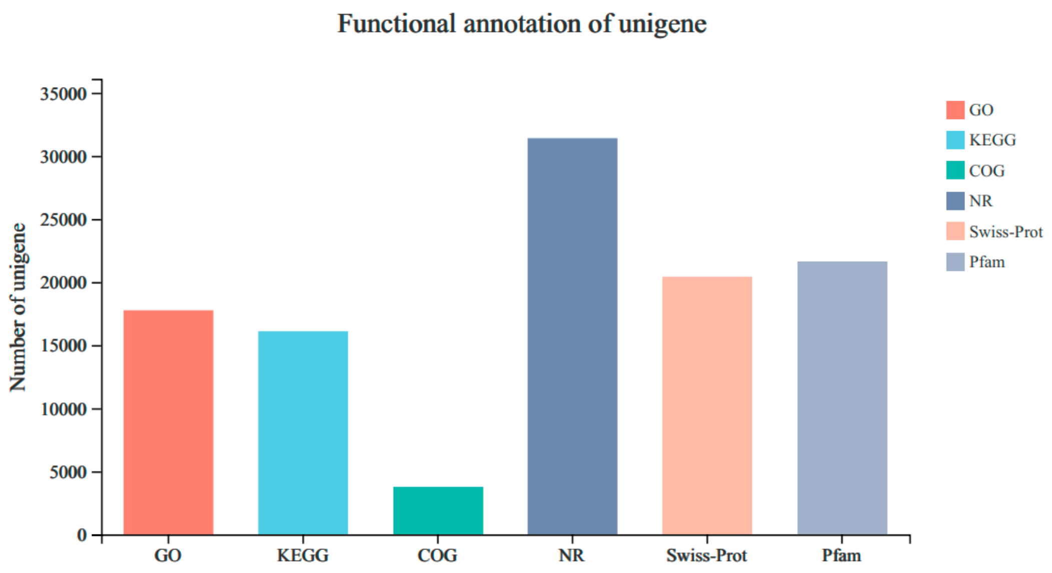

Figure S5 Six database annotation results.

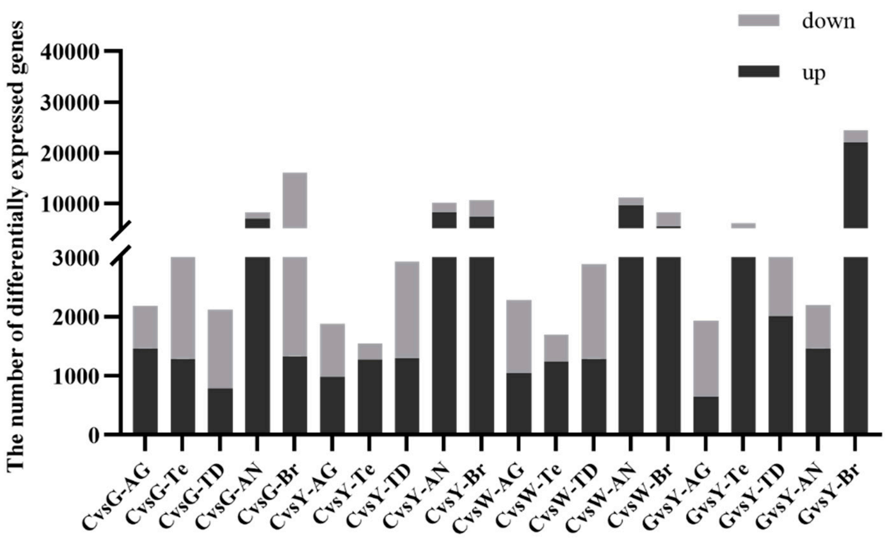

Figure S6 The number of differentially expressed genes. Br, brain; Te, testis; AG, androgenic gland; TD, testicular ducts; AN, abdominal nerve cord. C denotes the *P. clarkii* injected with saline (control group); G denotes the *P. clarkii* injected with 0.02 µg/g body weight GsiRNA (treated group); Y denotes the *P. clarkii* injected with 0.02 µg/g body weight YsiRNA (treated group); W denotes the *P. clarkii* injected with 0.02 µg/g body weight WsiRNA (negative control).

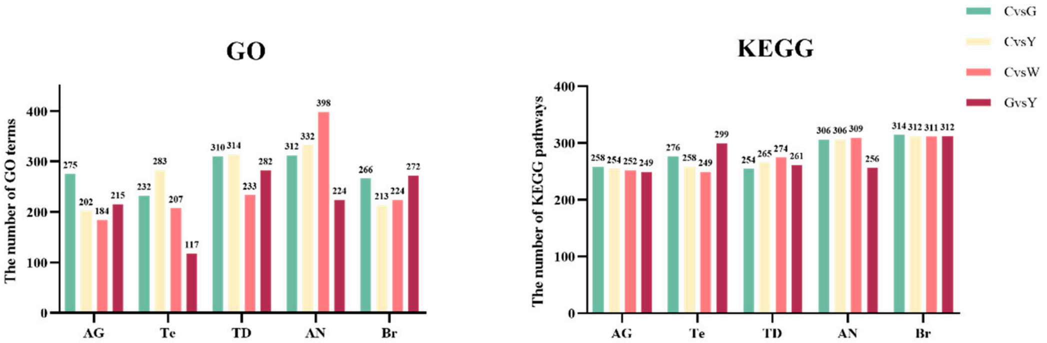

Figure S7 The number of GO terms and KEGG pathways enriched by differentially expressed genes between the groups with siRNA interference and control group. Br: Brain; Te: Testis; AG: Androgenic gland; TD: Testicular ducts; AN: Abdominal nerve cord. C *vs.* G denotes the number of differentially expressed genes in GsiRNA group (the *P. clarkii* injected with 0.02 µg/g body weight GsiRNA) compare to control group; C *vs.* Y denotes the number of differentially expressed genes in YsiRNA group (the *P. clarkii* injected with 0.02 µg/g body weight YsiRNA) compare to control group; C *vs.* W denotes the number of differentially expressed genes in WsiRNA group (the *P. clarkii* injected with 0.02 µg/g body weight WsiRNA) compare to control group; G *vs.* Y denotes the number of differentially expressed genes in GsiRNA group compare to YsiRNA group.

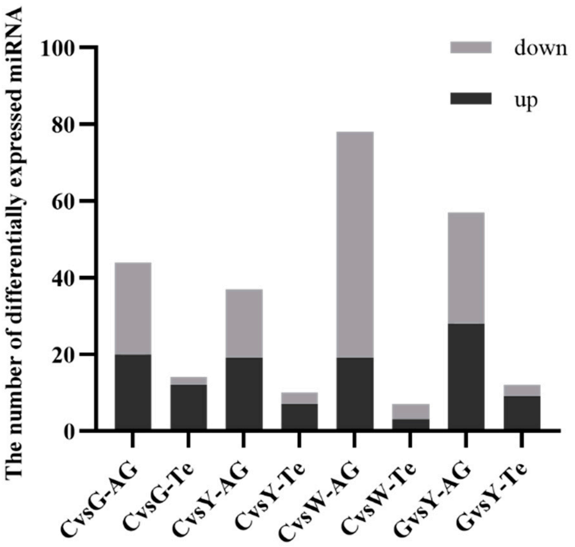

Figure S8 The number of differentially expressed miRNAs. Te, testis; AG, androgenic gland. C denotes the *P. clarkii* injected with saline (control group); G denotes the *P. clarkii* injected with 0.02 μg/g body weight GsiRNA (treated group); Y denotes the *P. clarkii* injected with 0.02 μg/g body weight YsiRNA (treated group); W denotes the *P. clarkii* injected with 0.02 μg/g body weight WsiRNA (negative control).

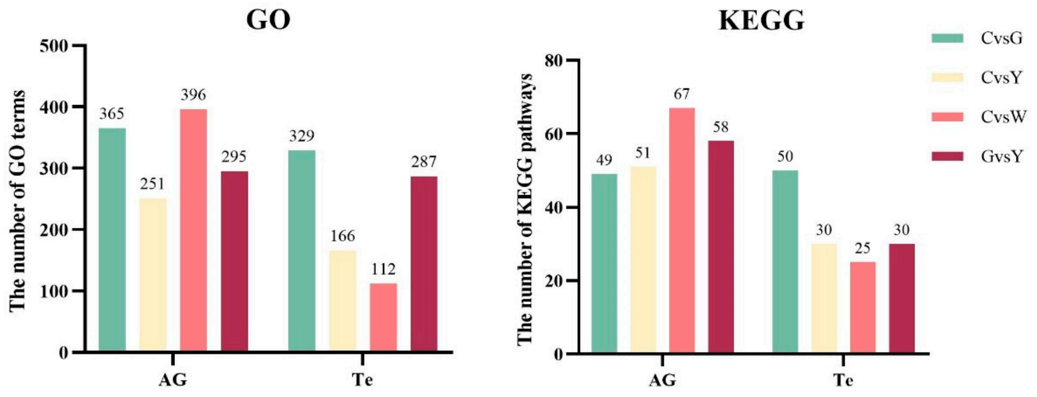

Figure S9 The number of GO terms and KEGG pathways enriched by target genes of differentially expressed miRNAs between the groups with siRNA interference and control group. Te: Testis; AG: Androgenic gland. C *vs.* G denotes the number of differentially expressed genes in GsiRNA group (the *P. clarkii* injected with 0.02 μg/g body weight GsiRNA) compare to control group; C *vs.* Y denotes the number of differentially expressed genes in YsiRNA group (the *P. clarkii* injected with 0.02 μg/g body weight YsiRNA) compare to control group; C *vs.* W denotes the number of differentially expressed genes in WsiRNA group (the *P. clarkii* injected with 0.02 μg/g body weight WsiRNA) compare to control group; G *vs.* Y denotes the number of differentially expressed genes in GsiRNA group compare to YsiRNA group.

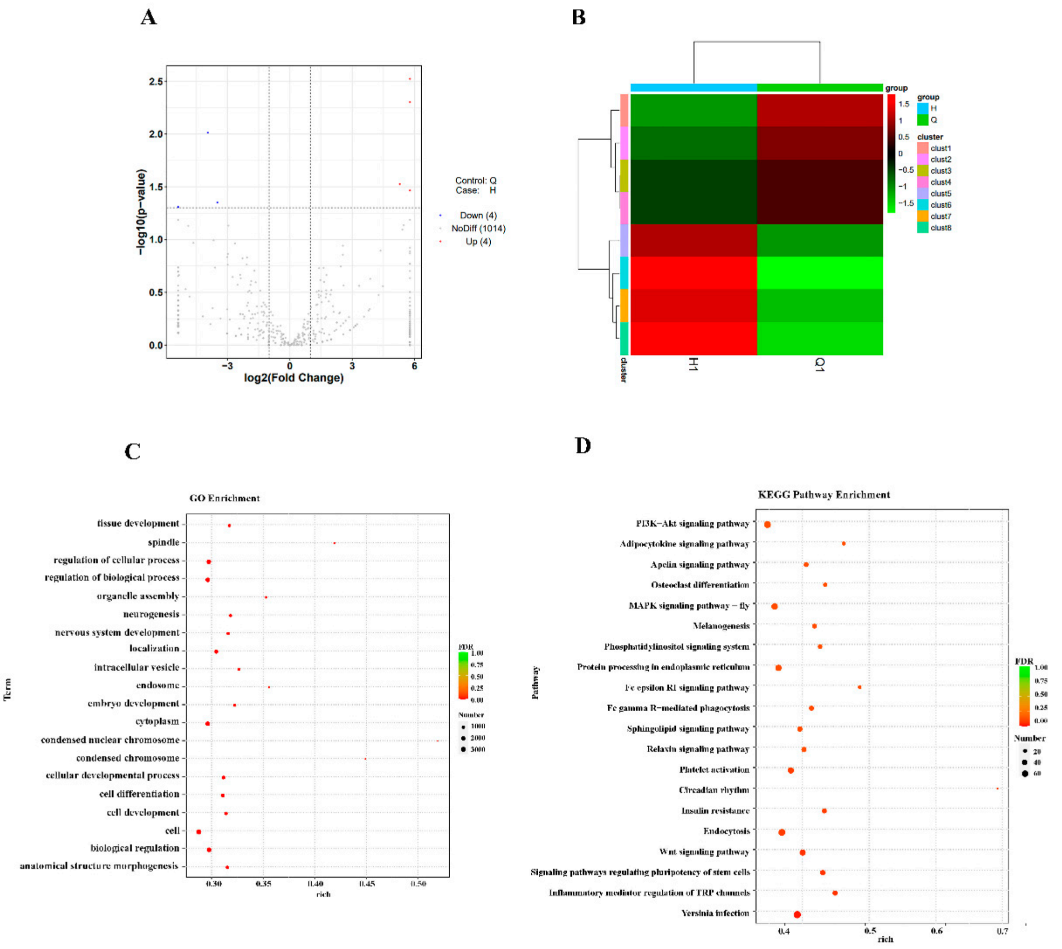

Figure S10 The volcano plot and heatmap of 8 differentially expressed miRNAs in the H group compared with Q group (p value < 0.05, and ± 2-fold change). **A**: The volcano plot of these miRNAs. The red dot, the upregulated miRNAs; the bule dot, the downregulated miRNAs; the gray dot, no differences in miRNA expression. **B**: The hierarchical clustering of 8 participants with these differentially expressed miRNAs. Red, upregulated expression of differentially expressed miRNAs; green, downregulated expression of differentially expressed miRNAs. Group Q, The exosomes in blood before androgenic gland ablation; Group H, The exosomes in blood after androgenic gland ablation. clust1: animal-mir-143-3; clust2: animal-mir-133-3; clust3: animal-mir-143-1; clust4: animal-mir-34-5; clust5: animal-mir-181-11; clust6: animal-mir-224-1; clust7: animal-mir-193-5; clust8: animal-mir-25-8. **C-D**: The functional analysis of the genes that are targeted by 8 differentially expressed miRNAs (p value < 0.05, and ± 2-fold change). C: GO enrichment analysis results. D: KEGG enrichment analysis results.
