# Supplementary material for: Two Short Repeats in the 5′ Untranslated Region of Insulin-like Androgenic Gland Factor in Procambarus clarkii (PcIAG) That Regulate PcIAG Expression
Source: Int J Mol Sci. 2022 Sep 7;23(18):10348. doi: 10.3390/ijms231810348 (PMC9499548; doi:10.3390/ijms231810348)
Supplement: Supplementary file 1 [file ijms-23-10348-s001.zip › Supplementary Materials Table.pdf]

Table S1 The sequencing information from twenty RNA-seq libraries

Group	Sample	Raw reads	Clean reads	Clean bases	GC content (%)
Saline	C_Br	45951774	44493612	6575583477	41.82
	C_Te	47727694	46289860	6818786566	44.35
	C_AG	43320146	42133980	6233671811	43.78
	C_TD	48710418	47193900	6955045983	45.12
	C_AN	50721078	48168226	7121823616	38.60
GsiRNA	G_Br	53326024	50175760	7387694234	43.62
	G_Te	45211756	43975644	6503216515	43.59
	G_AG	48324578	46944508	6926101460	43.88
	G_TD	51615270	48938120	7204100527	45.16
	G_AN	51846066	49183432	7270288914	41.25
YsiRNA	Y_Br	47759578	46404866	6858838846	41.25
	Y_Te	53992910	52304436	7679501471	44.54
	Y_AG	55035216	53548620	7897980582	44.34
	Y_TD	53910032	52227852	7642353110	46.72
	Y_AN	64242046	59852560	8825063896	41.95
WsiRNA	W_Br	40434376	39245818	5815832767	41.06
	W_Te	44094308	42893264	6319859768	43.47
	W_AG	57801844	56177204	8289700102	44.06
	W_TD	47151486	45608150	6677621508	46.81
	W_AN	48517978	47120280	6975148925	41.34

Note: Br, brain; Te, testis; AG, androgenic gland; TD, testicular ducts; AN, abdominal nerve cord. Saline denotes the *P. clarkii* injected with saline (control group); GsiRNA denotes the *P. clarkii* injected with 0.02 μg/g body weight GsiRNA (treated group); YsiRNA denotes the *P. clarkii* injected with 0.02 μg/g body weight YsiRNA (treated group); WsiRNA denotes the *P. clarkii* injected with 0.02 μg/g body weight WsiRNA (negative control).

Table S2 *De novo* assembly statistics of *Procambarus clarkii* transcriptome

	**unigenes/transcripts**
Total transcripts number	308120
Total unigenes number	184904
Largest length (bp)	21103
Smallest length (bp)	201
Average length (bp)	719.05
N50 length (bp)	1225
GC %	43.13

Table S5 Sex-related KEGG pathway

Group	Sex-related KEGG pathway in gonadal tissue (AG, Te, TD) and nervous tissue(Br, AN)	
C *vs*. G	AG, Te, TD	Ovarian steroidogenesis, Wnt signaling pathway, Progesterone-mediated oocyte maturation, Oocyte meiosis, Estrogen signaling pathway, GnRH signaling pathway, MAPK signaling pathway
	Br, AN	Ovarian steroidogenesis, Estrogen signaling pathway, Oocyte meiosis, GnRH signaling pathway, MAPK signaling pathway
C *vs*. Y	AG, Te, TD	Ovarian steroidogenesis, Wnt signaling pathway, Progesterone-mediated oocyte maturation, Oocyte meiosis, GnRH signaling pathway, MAPK signaling pathway
	Br, AN	Wnt signaling pathway, Oocyte meiosis, Progesterone-mediated oocyte maturation, Ovarian steroidogenesis, GnRH signaling pathway, MAPK signaling pathway
C *vs*. W	AG, Te, TD	Estrogen signaling pathway, Ovarian steroidogenesis, Progesterone-mediated oocyte maturation, Oocyte meiosis, Wnt signaling pathway, GnRH signaling pathway, MAPK signaling pathway
	Br, AN	Wnt signaling pathway, Progesterone-mediated oocyte maturation, Estrogen signaling pathway, Oocyte meiosis, GnRH signaling pathway, MAPK signaling pathway
G *vs*. Y	AG, Te, TD	Oxidative phosphorylation, Oocyte meiosis, Progesterone-mediated oocyte maturation、Wnt signaling pathway, Ovarian steroidogenesis, Estrogen signaling pathway, GnRH signaling pathway, MAPK signaling pathway
	Br, AN	Estrogen signaling pathway, Wnt signaling pathway, Oocyte meiosis, Progesterone-mediated oocyte maturation, Ovarian steroidogenesis, GnRH signaling pathway, MAPK signaling pathway

Table S6 Sex-related GO term in gonadal tissue

Group	Sex-related GO term in gonadal tissue (AG, Te, TD)
C *vs*. G	sex determination, female mating behavior, female gonad development, primary spermatocyte growth, male germ-line stem cell asymmetric division
C *vs*. Y	gonad development, male germ-line stem cell asymmetric division, entry into reproductive diapause, female mating behavior, ovarian follicle cell development, primary spermatocyte growth, ovarian follicle cell development, oocyte maturation, Wnt signaling pathway involved in dorsal/ventral axis specification, Wnt-activated receptor activity, coreceptor activity involved in Wnt signaling pathway, non-canonical Wnt signaling pathway
C *vs*. W	none
G *vs*. Y	male germ-line stem cell asymmetric division, entry into reproductive diapause, emale mating behavior, female gonad development, primary spermatocyte growth, ovarian follicle cell development, reproductive structure development, gonad development, Wnt signaling pathway involved in dorsal/ventral axis specification, coreceptor activity involved in Wnt signaling pathway, planar cell polarity pathway

Table S7 Sex-related differentially expressed genes

Unigene ID	Annotation	E-Value
TRINITY_DN33472_c4_g2	insulin-like androgenic gland hormone [*Procambarus clarkii*]	$6e^{-43}$
TRINITY_DN40696_c5_g1	hyperglycemic hormone-like peptide 2 precursor [*Procambarus clarkii*] (CHH A*)	$8.2e^{-43}$
TRINITY_DN33806_c3_g1	gonadotropin-releasing hormone II receptor isoform X1 [*Orussus abietinus*]	$2e^{-91}$
TRINITY_DN35788_c3_g3	sex-lethal 1 [*Macrobrachium nipponense*]	$9.7e^{-19}$
TRINITY_DN39703_c2_g1	DSX [*Sagmariasus verreauxi*]	$2.8e^{-40}$
TRINITY_DN22700_c0_g1	Dmrt11E [*Sagmariasus verreauxi*]	$2.1e^{-72}$
TRINITY_DN33221_c1_g1	insulin-like growth factor 2 mRNA-binding protein 2 isoform X4 [*Pseudomyrmex gracilis*]	$2e^{-140}$
TRINITY_DN39395_c0_g2	insulin-like receptor [*Macrobrachium rosenbergii*]	$4.1e^{-137}$
TRINITY_DN45278_c2_g1	membrane-anchored androgenic gland specific factor [*Cherax quadricarinatus*]	$2.1e^{-32}$
TRINITY_DN41831_c6_g1	transformer-2a [*Fenneropenaeus chinensis*]	$7.1e^{-57}$
TRINITY_DN39682_c0_g1	fem-1A [*Eriocheir sinensis*]	$4.1e^{-299}$
TRINITY_DN40273_c5_g1	fem1b [*Macrobrachium nipponense*]	0
TRINITY_DN31818_c1_g1	fem-1C [*Eriocheir sinensis*]	0
TRINITY_DN37030_c0_g2	fruitless [*Eriocheir sinensis*]	$5.5e^{-69}$
TRINITY_DN43249_c2_g1	SOX14B [*Eriocheir sinensis*]	$3.5e^{-32}$
TRINITY_DN46737_c0_g2	vitellogenin, partial [*Procambarus clarki*i]	$3e^{-199}$
TRINITY_DN34091_c1_g1	vitellogenin receptor [*Penaeus monodon*]	$4e^{-49}$
TRINITY_DN43157_c1_g1	cathepsin C [*Marsupenaeus japonicus*]	$3.3e^{-118}$
TRINITY_DN39227_c2_g4	Wnt4 [*Procambarus clarkii*]	$3.8e^{-120}$
TRINITY_DN41553_c1_g3	Wnt5 [*Litopenaeus vannamei*]	$4.6e^{-203}$
TRINITY_DN34662_c1_g1	RSPO1 [*Hyalella azteca*]	$5.1e^{-21}$
TRINITY_DN41966_c1_g1	forkhead box L2 [*Procambarus clarkii*]	$7.2e^{-144}$
TRINITY_DN36889_c2_g1	PIWI-1 [*Portunus trituberculatus*]	0
TRINITY_DN41724_c0_g1	PIWI-2 [*Portunus trituberculatus*]	$1.2e^{-279}$

Table S8 Sequencing information from eight small RNA-seq libraries

Group	Sample	Raw reads	Clean reads	Clean bases	GC content(%)	Useful reads(18nt-32nt)
Saline	C_AG	46855341	39142790	946754823	51.12	29239193
	C_Te	21284770	18625469	315261223	44.52	2931300
GsiRNA	G_AG	23793024	20095422	473437677	49.11	15464415
	G_Te	20616058	17507762	302133507	45.44	3398020
YsiRNA	Y_AG	36761577	27805859	617534698	50.59	19596428
	Y_Te	24316187	20984921	351860949	44.83	3341078
WsiRNA	W_AG	23339512	19574980	449873844	48.84	15204763
	W_Te	21667691	19133858	316293315	43.9	2430004

Note: Te, testis; AG, androgenic gland. Saline denotes the *P. clarkii* injected with saline (control group); GsiRNA denotes the *P. clarkii* injected with 0.02 μg/g body weight GsiRNA (treated group); YsiRNA denotes the *P. clarkii* injected with 0.02 μg/g body weight YsiRNA (treated group); WsiRNA denotes the *P. clarkii* injected with 0.02 μg/g body weight WsiRNA (negative control).

Table S11 The number of target genes of known miRNA and novel miRNA in gonadal tissue

Type	miRNA	miRNA with Target	Target
**Known miRNA**	300	295	863
**Novel miRNA**	374	332	1212
**Total**	674	627	1536

Table S13 Sex-related GO term in gonadal tissue

Group	Sex-related GO term in gonadal tissue (AG, Te)
C *vs*. G	ovarian follicle cell stalk formation, female germ-line cyst encapsulation, cytoplasmic transport, nurse cell to oocyte, ovarian follicle cell migration
C *vs*. Y	negative regulation of canonical Wnt signaling pathway, regulation of canonical Wnt signaling pathway
C *vs*. W	none
G *vs*. Y	none

Table S14 The number of known miRNA and novel miRNA in exosomes

Group	Known miRNA	Novel miRNA
Q	689	54
H	666	33

Note: Group Q, The exosomes in blood before androgenic gland ablation; Group H, The exosomes in blood after androgenic gland ablation.

Table S18 Differentially expressed genes associated with Wnt signaling pathway

Unigene ID	KO name	gene	differentially expressed miRNAs in exosomes of blood
TRINITY_DN48621_c0_g1	K04345	Protein kinase A (*PKA*)	miR-181/ miR-25
TRINITY_DN23239_c0_g2<br>TRINITY_DN42544_c3_g5<br>TRINITY_DN17749_c0_g3<br>TRINITY_DN17749_c0_g1<br>TRINITY_DN2705_c0_g1<br>TRINITY_DN17749_c0_g2	K02105	β-catenin (*CTNNB1*)	miR-224/ miR-181
TRINITY_DN40593_c1_g2<br>TRINITY_DN34662_c1_g1	K19471	R-spondin 1 (*RSPO1*)	miR-143/miR-181/miR-193/miR-224/miR-34/ miR-25
TRINITY_DN41553_c1_g3	K00444	Wnt Family Member 5A (*Wnt5*)	miR-224/miR-25
TRINITY_DN40370_c3_g2	K06268	Serine/Threonine-protein phosphatase 2B regulatory subunit (*CaN*)	miR-181/miR-143/miR-224
TRINITY_DN45094_c3_g1	K04440	Mitogen-activated protein kinase (*JNK*)	miR-181/miR-193
TRINITY_DN43997_c5_g1<br>TRINITY_DN45373_c4_g1	K04427	mitogen-activated protein kinase kinase kinase 7 (*TAK1*)	miR-181
TRINITY_DN34140_c3_g3<br>TRINITY_DN34140_c3_g2<br>TRINITY_DN34140_c3_g1	K04468	Nemo like kinase (*NLK*)	/
TRINITY_DN30517_c4_g5	K03362	F-box and WD-40 domain protein 1/11 (β-*Trcp*)	miR-143/miR-224/miR-34/ miR-25
TRINITY_DN33452_c1_g3<br>TRINITY_DN33452_c1_g1	K03094	S-phase kinase-associated protein 1 (*SKP1*)	miR-143/miR-34
TRINITY_DN38911_c0_g2<br>TRINITY_DN38911_c0_g1	K03347	Cullin 1 (*CUL1*)	miR-143/ miR-181/miR-224
TRINITY_DN20157_c0_g1<br>TRINITY_DN36344_c1_g3	K03868	E3 ubiquitin-protein ligase RBX1 (*RBX1*)	/
TRINITY_DN37425_c0_g8<br>TRINITY_DN37425_c0_g1<br>TRINITY_DN45062_c4_g1	K05858	Phosphatidylinositol phospholipase C (*PLC*)	miR-181/miR-34/ miR-25
TRINITY_DN40174_c5_g1	K02677	Classical protein kinase C alpha type (*PKC*)	miR-181/miR-193

Table S19 Nucleotide sequences of primers used for *PcIAG* cloning

Primer name	Primer sequences（5’ – 3’）	Purpose
PcIAG -F1	CCTAAATCAACAATGCTTGAAATAG	Full-length DNA cloning of *PcIAG*
PcIAG-R2	CGTTGTATTCGTTGAAGGTGAG	
PcIAG-F2	GTCAGTCTGCATTTTCGTCAA	
PcIAG-R2	GAAGGTAGTTGTGCTTTCTGGGTTC	
PcIAG-F3	ATACTGCAGGTGCTGGTGGCGGTGT	
PcIAG-R3	TGTGTCTTGACGAAGGTGTGA	
Qi-F4	CGTGTGAGGTGCAAACTATGAAAT	Promoter sequences cloning of *PcIAG*
Qi-R4	CAGTTTCAGTAATGTTTGAGTGAGC	
PcIAG-F	CGGCACTCCCCTGTCCCAACT	Amplification of *PcIAG* repeat regions
PcIAG-R	TTCAGTAATGTTTGAGTGAGCATCG	
PcIAG-qF	CAGCAGCCACTGTGACGAT	For checking the siRNA interference effect
PcIAG-qR	GATGTCCTCAGCGGGGTGG	
18s-F	TATACGCTAGTGGAGCTGGAA	Housekeeping genes
18s-R	GGGGAGGTAGTGACGAAAAAT	
